# Unravelling trait-mediated effects of genotype and companion planting system on oilseed rape performance

**DOI:** 10.3389/fpls.2026.1878300

**Published:** 2026-07-30

**Authors:** Laurie Magnin, Ivan Hiltpold, Alice Baux, Alain Fortineau, Alexandra Jullien

**Affiliations:** 1 Entomology and Nematology, Agroscope, Nyon, Switzerland; 2 Cultivation Techniques and Varieties in Arable Farming, Agroscope, Nyon, Switzerland; 3 Université Paris-Saclay, Institut national de recherche pour l’agriculture, l’alimentation et l’environnement (INRAE), AgroParisTech, UMR ECOSYS, Palaiseau, France

**Keywords:** ecophysiology, intercropping, irradiance, phylloclimate, plant architecture, secondary metabolites

## Abstract

**Introduction:**

Functional traits, such as morphology, phenology, and chemical defenses, are key factors of plant–plant and plant–environment interactions and are central to predicting ecosystem responses in managed agrosystems. Growing oilseed rape (OSR, *Brassica napus*) with legumes, as a key agroecological lever, can enhance yield and sustainability. However, the trait-based mechanisms underlying these benefits remain unclear.

**Methods:**

We investigated the morphological development, phenology, and glucosinolate defense traits of three OSR varieties grown as monocrops or grown with faba beans (FB, *Vicia faba*) companion plants under controlled glasshouse and two-year field conditions to explore competition and/or facilitation processes. We further characterized the crop microclimate (temperature, light quantity, light quality) and monitored insect pests to link OSR trait responses with agronomic outcomes.

**Results:**

Growing OSR with FB companion plants consistently reduced OSR leaf development both in the greenhouse and in the field due to reduced photosynthetically active radiation under the FB canopy, revealing light-driven plasticity in early vegetative traits. At the reproductive stage, trait expression diverged across environments: in glasshouse conditions, excessive FB competition suppressed OSR reproductive development, while in the field, winter froze FB stems, which alleviates shading, triggering compensatory stem elongation, increased branching, and enhanced yield of OSR. Yield gains were thus mediated by morphological and phenological trait adjustments rather than direct pest suppression. Glucosinolate concentrations and profiles were strongly shaped by environment and variety, but companion planting notably decreased OSR total glucosinolates after winter in the field, indicating context-dependent defense trait responses.

**Discussion:**

Our findings highlight how environmental context and varietal identity govern the expression of aboveground functional traits in OSR–FB companion planting systems. Beyond its contribution to pest regulation and despite contrasting effects on plant traits throughout development, OSR–FB companion planting demonstrated agronomic viability by promoting OSR adaptive responses that maintained productivity under field conditions.

## Graphical abstract

**Figure:**
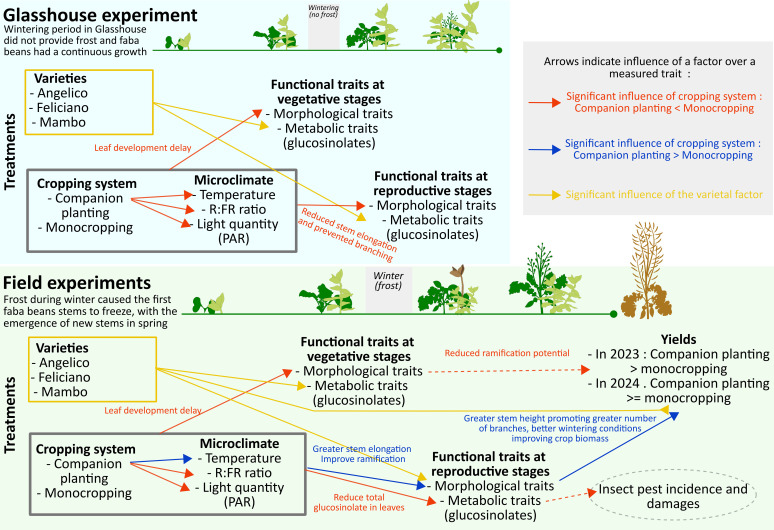

## 1 Introduction

Functional traits of plants, such as morphology, phenology, and chemical defenses, are key factors of plant-environment and plant-plant interactions (Garnier and Navas, 2012). With the increase of agroecosystem complexity, this approach is central to predict its responses in terms of yield but also in terms of sustainable criteria such as crop protection, allowing input reductions (Moreau et al., 2025; Wood et al., 2015). Oilseed rape (OSR, *Brassica napus*) is one of the most widely cultivated oil crops, valued for its high oil content and protein-rich by-products (Carré and Pouzet, 2014). However, OSR production faces significant agronomic challenges, including pest pressure, weed competition, and environmental sustainability concerns (Charles et al., 2020; Ortega-Ramos et al., 2021). Growing OSR with companion plants has emerged as a promising strategy to optimize resource use. Companion planting system (CPS) refers to the practice of cultivating a companion crop alongside a cash crop in the same field, fostering close interaction throughout a significant portion of the cash crop’s life cycle (Gardarin et al., 2022; Magnin et al., 2026; Seimandi-Corda et al., 2024).

Growing OSR with legume companion plants has been shown to enhance soil fertility, reduce weed growth, and potentially mitigate pest incidence while maintaining or improving yield. Faba Bean (FB, *Vicia faba*) is a favorable candidate as a companion plant as it is resistant to competition, has a minimal impact on OSR, and fixes large amounts of nitrogen (Bousselin et al., 2021; Verret et al., 2017). According to the literature, different agronomic performances are observed depending on whether the CPS is additive (the FB is added to the crop, and the density of plants is increased compared to the pure crop) or substitutive (FB is substituted for OSR, the density of plants is identical to the pure crop). In an additive CPS, frost-sensitive FB enabled a reduction of 20 to 40 kg·ha^-1^ in nitrogen fertilizer use without compromising OSR yield and decreased weed abundance by 20–75% compared to monocropped OSR, with soil nitrogen availability playing a key role in this effect (Lorin et al., 2015, 2016). Under a substitutive design, OSR-FB CPS reduced weed biomass, which was negatively correlated with crop biomass (Dayoub et al., 2022).

The OSR-FB CPS has also been found to reduce plant damage caused by the cabbage stem flea beetle (*Psylliodes chrysocephala*) adults in autumn, as well as its larval infestation (Breitenmoser et al., 2020; Breitenmoser et al., 2022; Coston et al., 2022; Emery et al., 2021; Magnin et al., 2025). Additionally, growing OSR with frost-sensitive or frost-resistant FB decreases the number of the rape stem weevil (*Ceutorhynchus napi*) oviposition punctures and the number of pollen beetle (*Brassicogethes aeneus)* per inflorescence compared to monocropped OSR (Breitenmoser et al., 2022; Magnin et al., 2025). The yield of OSR grown with FB in additive CPS was found to be improved by 0.16 t.ha−1 on average in an extensive study across 79 fields (Verret et al., 2017). However, the determinants of the CPS effects and its agronomic performance remain unclear. It is assumed that a better understanding of the functional traits involved in the impact of CPS on the insect resistance of OSR and ultimately the stability or improvement of its yield will help to identify these determinants.

The winter OSR follows a specific growth cycle, starting with vegetative development in autumn, during which it forms a rosette structure to withstand winter. As winter transitions into spring, the plant enters its reproductive phase, characterized by stem elongation, main stem branching, flowering, and pod filling (Pinet et al., 2015). Throughout this cycle, environmental conditions will trigger physiological responses shaping the plant’s functional traits, encompassing both morphological, phenological, and metabolic traits (Jullien et al., 2009, 2011). These traits influence pest resistance and yield formation, ultimately affecting crop success (Pigliucci, 2005). Oilseed rape demonstrates significant morphological plasticity across its growth stages, which is modulated by both genotype and environmental factors (Jullien et al., 2009, 2011; Pinet et al., 2015). One key metabolic trait is the glucosinolate-myrosinase system, a well-known defense mechanism in Brassicaceae, which may help protect the plant from various generalist pests and pathogens (Giamoustaris and Mithen, 1995). However, specific glucosinolates (GLS) and their breakdown products can also act as attractants and feeding stimulants for specialist insects adapted to brassicaceous hosts (Grosjean et al., 2024; Magnin et al., 2026; Renwick et al., 2006).

At the vegetative stage, CPS may affect biomass allocation, leaf development, and defense compound production in OSR, influencing insect resistance and yield formation. Leaf production in autumn plays a crucial role in determining the potential number of stem ramifications, which is a key yield component in OSR (Diepenbrock, 2000). Studies have shown that OSR grown in substitutive or additive CPS with FB have greater biomass compared to monocropped OSR (Dayoub et al., 2022; Lorin et al., 2015). Increased crop ground cover during the period of *P. chrysocephala* immigration may reduce the adult feeding damage, while larval infestation is correlated to OSR biomass (Hausmann et al., 2023; Seimandi-Corda et al., 2024). Additionally, both feeding damage and larval infestation by *P. chrysocephala* have been associated with GLS leaf content (Bartlet et al., 1994; Grosjean et al., 2024; Magnin et al., 2026; Wallsgrove et al., 1999; Wittstock et al., 2003). Notably, foliage GLS levels in *B. oleracea* have been observed to decrease in response to CPS (Björkman et al., 2008).

At the reproductive phase, CPS may influence stem elongation dynamics, ramification formation (Pinet et al., 2015), and OSR defense compound production, potentially affecting spring-active insect pests and yield formation. The stem height of OSR was found to be negatively correlated with the number of oviposition punctures by *C. napi* (Ballanger, 1987; Büchi, 1996; Schaefer-Koesterke et al., 2017). For *B. aeneus* plant functional traits —including growth stage (Hervé et al., 2017), GLS content, and plant size— can influence infestation levels and damage (Cook et al., 2006; Rusch et al., 2013; Seimandi-Corda et al., 2019).

Companion planting may also modify the above-ground growing environment, influencing OSR functional traits through various indirect mechanisms. The density and repartition of the plants may impact the crop microclimate with the light quantity and quality, and the temperature in the canopy. In shaded environments, leaf optical properties reduce blue and red photons while increasing far-red photons, lowering irradiance and the red/far-red (R/FR) ratio. This alters plant competition and can affect crop productivity (Casal, 2013). Plants detect low R/FR ratios from neighboring plants and respond morphologically before shading occurs (Ballaré et al., 1987). Early shade signals trigger shade avoidance syndrome, leading to stem and petiole elongation, leaf reorientation, accelerated leaf senescence, and an increased shoot/root ratio; however, differing effects have been observed on the floral branches in OSR (Fortineau et al., 2021; Rondanini et al., 2014). The R/FR ratio is known to influence glucobrassicin and gluconasturtiin production in *B. oleracea* (Renner et al., 2021); both GLS are also present in *B. napus*. This suggests that light quality may similarly affect GLS biosynthesis in OSR. Canopies of CPS may have different under-canopy temperatures compared to monocropped systems. For example, intercropping FB with wheat results in a warmer canopy temperature compared to monocropped FB (Ma et al., 2023). These temperature differences may influence the morphological development of plants by altering the phylloclimate, which is the environment directly perceived by each individual aerial organ within a plant population (Chelle, 2005).

Our study examines the impact of OSR-FB additive CPS on above-ground ecophysiological functional traits possibly linked to pest resistance and yield across various OSR cultivars under non-limiting nitrogen conditions. We assume that CPS will have an impact on vegetative development of OSR, on morphological characteristics in reproductive stages, and on secondary metabolite defense compounds such as GLS. Through the monitoring of microclimate characteristics, we aim to give insight into the mechanisms at work in the OSR-FB CPS, which may lead to changes in ecophysiological traits. First, a glasshouse (GH) experiment was conducted to closely monitor the development of OSR grown with FB plants under controlled conditions, without insect interference. This was followed by a two-year field trial to validate the GH model under real conditions, where natural insect pests were present, and to perform yield analysis. In our study, we selected a frost-resistant variety of winter FB as a companion plant. This setup was designed to assess the impact of the companion plants on the spring-active insect pests of OSR. It also provided an opportunity to evaluate OSR performance grown with companion plants that remained in the field following a mild winter, when frost did not fully eliminate them.

## 2 Material and methods

### 2.1 General setup

**Figure 1:**
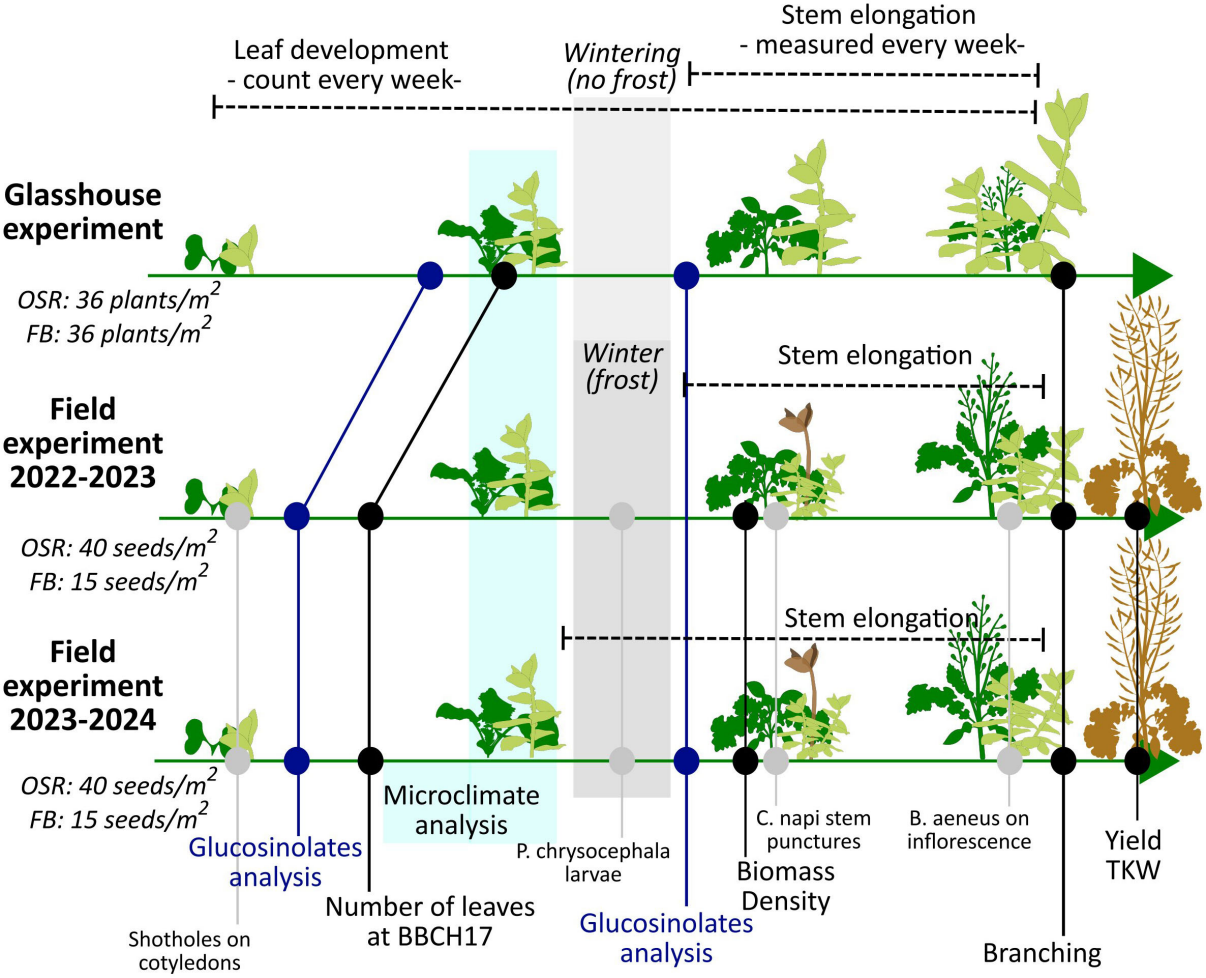
Schematic overview of the experimental conditions and data collected from the three experiments. For each experiment, three oilseed rape (OSR) varieties—Angelico (Limagrin, FR), Mambo (Euralis Semences, FR), and Feliciano (KWS, DE)—were either grown as monocrops or grown with winter faba beans (FB, var. Augusta, UFA, Switzerland). For clarity, only the companion planting system for one OSR variety is represented. Oilseed rape plants are shown in dark green, while FB is depicted in light green. Under glasshouse conditions, the FB did not freeze and showed continuous growth. In contrast, under field conditions, frost damaged the first FB stem during winter (depicted with a brown stem), and the FB regrew from its base under the OSR canopy. Grey dots show the data collected on pest incidence and damage, chronologically done by the cabbage stem flea beetle (*Psylliodes chrysocephala*), the rape stem weevil (*Ceutorhynchus napi*), and the pollen beetle (*Brassicogethes aeneus*). Blue dots show the OSR leaf sampling for glucosinolates analysis. Black dots show the data collected on morphological traits of OSR plants, and the light blue shade shows the microclimate monitoring.

These experiments took place in 2022 in glasshouse (GH) conditions and in open field conditions, the 2022–2023 and the 2023–2024 cropping seasons (**Figure 1**) in Switzerland (Agroscope station, Nyon). Three varieties of OSR (1- Angelico, Limagrin, FR; 2- Mambo, Euralis Semences FR; 3- Feliciano, KWS, DE) were either grown alone as a monocrop or with a frost-resistant winter FB (var. Augusta, UFA, CH) in the companion planting system (CPS).

#### 2.1.1 Glasshouse

Ninety-litter plastic containers (60 × 40 × 42 cm; Ref EG 64/42, AUER Packaging, Germany) were used as pots to cultivate nine OSR plants (36 plants.m-2), either alone or with the addition of nine FB plants (36 plants.m-2), to simulate different canopy structures. Each combination of OSR cultivar and cropping system (monocropping or CPS) was replicated five times. The OSR was sown in three 1 cm–deep rows spaced 20 cm apart, and in the CPS, FB was sown in three 5 cm–deep rows between the OSR rows. Plants were grown on a horticultural substrate (TYPical, Bryl, France) supplemented with 150 g of slow-release fertilizer (Osmocote Exact, REF.95876, Landi, Switzerland). Glasshouse growing conditions (temperatures, photoperiod, and lighting supplementation) are detailed in box 1 of the supporting information.

#### 2.1.2 Field experiment

Oilseed rape was sown in 24 m² plots (2 × 12 m) in both seasons. In 2022–2023, the field was arranged in three semi-randomized blocks, with each variety × cropping system replicated three times. Winter OSR was sown on 22 August 2022 (40 seeds m^-2^; 21 cm row spacing), and FB (15 seeds m^-2^) was undersown randomly on the same date before OSR sowing. In 2023–2024, the field was organized in four semi-randomized blocks, with each treatment replicated four times. Winter OSR was sown with a newly available seed drill, designed for sowing faba beans in the inter-row, on 18 August 2023 (40 seeds m^-2^; 30 cm row spacing), and FB (15 seeds m^-2^) was sown between OSR rows on the same date, allowing a more homogeneous spatial arrangement of the plants.

The fields had both sandy clay loam soil and received 50 m³/ha of cattle manure in early August before sowing, followed by nitrogen supplementation in February (75 kg/ha) and March (75 kg/ha), along with 30 units of sulfur in March (200 kg/ha Kieserite, Landor, Switzerland). A pre-emergence herbicide (3 L/ha Devrinol Plus, Staehler, Switzerland) was applied five days after sowing. Two additional herbicides were used, one for cereal volunteers (1.5 L/ha Fusilade Max, Syngenta, Switzerland) after OSR emergence in early September, and one for monocotyledonous weeds (1.4 L/ha Arlit, Omya, Switzerland) in November. No insecticides nor fungicides were applied.

### 2.2 Data collection

#### 2.2.1 Data collection on morphological traits

The data collected on the GH and field experiments are summarized in **Figure 1**, and the replication statement table can be found in **Supplementary Table 1**.

##### 2.2.1.1 Leaf development (glasshouse and field)

In the glasshouse experiment, unfolded leaves were counted weekly on four plants per pot. This was done from the phenological stages BBCH 13 (three unfolded true leaves) to BBCH 50 (inflorescence emergence) (Lancashire et al., 1991), before and after wintering (n = 20 plants per treatment).

In the field experiments, the number of leaves per plant was counted when OSR was estimated to have seven true leaves unfolded (BBCH 17) and before any true leaves started to fall. The total number of true leaves was counted for three series of five adjacent OSR plants per plot (n = 45 plants in 2022 and n=60 in 2023 plants for each treatment). In the 2023–2024 cropping season, the number of leaves was counted a second time in mid-November following the same method.

##### 2.2.1.2 Plant density and biomass (field)

In fields for both years, the number of OSR plants and FB plants was counted in mid-February on a square meter randomly chosen for each plot.

Aboveground biomass was measured at the end of the winter vegetative phase to assess the effects of faba bean on OSR vegetative growth and to complement measurements of morphological traits collected throughout crop development. This sampling stage was also selected to coincide with the assessment of *P. chrysocephala* larval infestation, allowing both responses to be evaluated under comparable conditions. See section 2.2.2.2 of this material and method for the procedure details and sampling condition.

##### 2.2.1.3 Stem elongation (glasshouse and field)

The stem elongation was measured from the center of the apical bud to the soil substrate for all experiments. In the glasshouse experiment, the stem elongation was measured every week from the end of wintering to the blooming period on four plants per pot (n =20 plants for each treatment).

In the field experiments, stem elongation was monitored on three sets of five adjacent OSR plants per plot. In 2022–2023, measurements began on 1 February, at the onset of post-winter growth, and were taken every two weeks until flowering. In 2023–2024, elongation started before winter; the first measurement was taken in mid-November, followed by weekly measurements from mid-February to early April.

##### 2.2.1.4 Branching (glasshouse and field)

In the GH experiment, the number of branches was counted at the end of the blooming period (BBCH 67). The number of branches was counted on four plants per pot when accessible to provide at least the measure on 12 plants per treatment.

In the 2022–2023 cropping season, the number of branches was counted at the beginning of ripening (BBCH 80). During the 2023–2024 cropping season, the number of branches was counted at the beginning of the blooming period (BBCH 61) on three series of five adjacent OSR plants per plot, as the plants were more developed and did not allow field entry afterward without causing damage.

##### 2.2.1.5 Faba beans monitoring

Faba bean development was visually assessed throughout the entire crop cycle in all experiments. The relative position of the FB canopy compared with the OSR canopy (i.e., below or above the OSR canopy) was recorded, together with the physiological status of FB plants (frost-damaged or actively growing).

#### 2.2.2 Data collection on insect pest incidence (field)

##### 2.2.2.1 Cabbage flea beetle (*Phyllotreta* spp.) feeding shot-holes on cotyledons

The number of flea beetle feeding shotholes, characterized by “shot-gun” holes on OSR plants’ cotyledons, was counted when two true leaves were unfolded (BBCH 12). In both years, the crop reached this stage before the immigration of *P. chrysocephala*, as monitored by the yellow water trap. Consequently, the damage was attributed to cabbage flea beetle belonging to the *Phyllotreta* genus caught in the traps (Breitenmoser, 2019). In the 2022–2023 season, the number of feeding shot-holes was counted on the 9th of September on two series of five adjacent OSR plants per plot (n = 30 plants per treatment). In the 2023–2024 season, it was counted on the 11th of September on three series of five adjacent OSR plants per plot (n = 60 plants per treatment).

##### 2.2.2.2 Cabbage stem flea beetle (*Psylliodes chrysocephala*) larval infestation

At the end of winter vegetative phase (BBCH 30), the above-ground parts of sampled plants were cut at the stem base, just below the soil surface. In 2022-2023, five consecutive plants per plot were sampled, while in 2023-2024, three sets of three plants were taken. Leaf blades were removed, and plants were pooled—five per group in 2022–2023 and three per group in 2023-2024. They were then placed on Berlese traps (Seimandi‐Corda et al., 2022). Larval extraction lasted three weeks until plants were fully dry. Plant weight was measured again afterward allowing to extrapolate the plant biomass (section 2.2.1.2).

##### 2.2.2.3 Rape stem weevil (*Ceutorhynchus napi*) oviposition punctures

Oviposition damage by *C. napi*, identified by punctures enclosed in white mucus on the OSR main stem, was counted on three sets of five adjacent OSR plants per plot (n= 45 plants in 2022–2023 and n= 60 plants in 2023-2024). Oviposition punctures were counted on the 1st of March in the 2022–2023 season and the 19th of March in the 2023–2024 season (BBCH 32 in 2023, BBCH 34 in 2024).

##### 2.2.2.4 Pollen beetle (*Brassicogethes aeneus*) abundance

The number of *B. aeneus* per main inflorescence was counted by the beating method on three series of five adjacent OSR plants per plot. Each inflorescence was individually shaken in a small plastic bowl (20 cm diameter, 10 cm deep), and *B. aeneus* were counted before being released. This was done on the 21st of March 2023 and the 22nd of March 2024 before the first flowers bloomed (BBCH 57).

#### 2.2.3 Yield (field)

The OSR was harvested on the 10th of July 2023 and on the 4th of July 2024 (BBCH 89-97), with an SP2100 experimental harvester (Baural, Champigny-en-Beauce, France) as described by Breitenmoser et al. (2022). Net grain yield (dried to the standard 6% relative moisture) was recorded. Technical limitations restricted the harvest to OSR only.

##### 2.2.3.1 Thousand kernel weights (field)

Thousand kernel weight (TKW, g) was measured at the plot level with a Contador seed analyzer (PFEUFFER, Kitzingen, Germany). The seed density was calculated by dividing the yield (kg.m-2) by the TKW (kg).

#### 2.2.4 Data collection on defense traits - glucosinolates (glasshouse and field)

In the GH experiment, leaves from two plants per pot were sampled for GLS analysis before wintering (n= 10 leaf samples per treatment). After wintering, samples were taken from two other plants following the same procedure (n= 10 per treatment). In the 2022–2023 cropping season, the leaves from three randomly chosen OSR plants per plot were sampled for GLS analysis on the 11th of November (n= 9 per treatment). In the 2023–2024 cropping season, the leaves of three consecutive plants were pooled for one sample. Three samples per plot were taken (n= 12 per treatment) on the 3rd of October and on three new samples after winter, the 20th of February, following the same procedure. For each sampling date and experiment, the youngest unfolded leaf (including petiole) was sampled on selected plants. They were frozen in liquid nitrogen and kept at -80 °C. Metabolite extraction was performed as described in Magnin et al., 2026. Samples were stored at -80 °C until the HPLC-MS analysis (injection volume: 0.25 µL, resolution mode; as described in Glauser et al., 2012). Post-winter GLS analyses were performed only for the 2023–2024 field experiment; therefore, no spring GLS data were available for the 2022–2023 season.

#### 2.2.5 Data collection on the cropping systems microclimate (GH and Field)

##### 2.2.5.1 Temperature within the canopy

Two automated dataloggers were assembled to measure air temperature locally under the canopy in the GH and in the fields. Each consists of a CR10X datalogger and a measurement port multiplexer AM32 (Campbell, Logan, USA) to which are connected the temperature sensors made from 0.2 mm diameter T-type thermocouple cable (TC-direct, Dardilly, France) and a reference thermistor 10TCRT (Campbell).

All the sensors from both acquisition chains were calibrated by comparison with a reference 1523 temperature sensor (Fluke, Utah, USA) in a 7103 thermoregulated bath (Fluke, Utah, USA) to define an offset and a slope. These constants were integrated into the data acquisition program to obtain corrected values with an uncertainty of < +/-0.1 °C.

Data acquisition was programmed to scan sensor readings every 30 s and calculate the average value for each sensor over a time step of 15 min. The clock was checked regularly to correct any drift. Under experimental conditions, the sensors were placed under the canopy at 10 cm above the ground and out of the canopy for temperature control (at least three sensors).

In the GH experiment, sensors were placed individually on five pots per side of the GH (n = 10 per cropping system). The sensors were placed five days prior to the wintering period (BBCH 7), and temperature was collected continuously for five days. In the field experiments, only one CR10X was used to monitor temperature on two blocks. Each plot of those two blocks was monitored by a sensor (n = 6 per cropping system) during 8 days in November (starting from the 07.11.2022 in the 2022–2023 season and from the 07.11.2023 in the 2023–2024 season).

##### 2.2.5.2 Light quantity and quality with a spectroradiometer (glasshouse and field)

The light quantity (Photosynthetic active radiation - PAR) along a vertical profile of the canopy was monitored through a movable set-up composed of a pole and two sensors, an incident one and a transmitted one. The incident sensor (Li-COR LI-190R quantum, Li-COR, USA) linked to an automated data acquisition station (CR10, Campbell, USA) was fixed on a pole at 1.5 m to measure the incident light reaching the canopy. These measurements were automatically logged every 30 s. A second light sensor, a spectroradiometer (CSS-45, Gigahertz Optik GmbH, Germany) linked to a display unit (CSS-D, Gigahertz-Optik, Germany) was placed on a vertical translation support along the pole, allowing it to measure transmitted light quality at various heights along the vertical profile of the canopy.

In the glasshouse experiment, to take account of artificial lighting only, which accounted for most of the radiation received by the plants (Box 1 in supp. information), and to ensure repeatability of radiation characterization, the measurements of the light quality (red/far-red ratio) and quantity were carried out without sunlight, *i.e*., at night. Measurements were taken on the 7th of June at BBCH 31. The pole was placed equidistant from the OSR and FB rows in the pots. The CSS-45 sensor was first placed on the ground for the initial measurement, then readings were taken every 10 cm along the pole up to 140 cm. Two sets of measurements were taken for each sampled pot (n= 14 per cropping system). The percentage of transmitted PAR through the canopy was calculated by dividing the PAR measured under the canopy (CSS-45 sensor) by the incident PAR above the canopy (LiCOR sensor) and multiplying by 100. The corrected PAR was then obtained by multiplying this percentage by the average PAR of multiple localizations in the GH on a cloudy day.

In the 2023–2024 field experiment, the light quality and quantity were measured on the 23rd of November at midday on a sunny day (BBCH 31). The pole was placed equidistant from the OSR and FB rows in the plot. The CSS-45 sensor was first placed on the ground for the initial measurement, then readings were taken above the OSR canopy and the FB canopy (n=9 per cropping system). For each set of spectroradiometer measurements, the height of both the OSR and FB canopies was measured using a ruler.

### 2.3 Statistical analysis

All the analysis was performed in R version 4.4.1 (R Core Team, 2024).

#### 2.3.1 Statistical analysis of the morphological traits, pest incidence, and yield

The effects of variety, cropping system, and their interaction were tested separately within each experimental setup (GH, Field 2022–2023, Field 2023–2024). Depending on the data type and distribution, linear mixed-effects models (lme4) (Bates et al., 2015) or generalized linear mixed models (glmmTMB) (Brooks et al., 2017) were applied, including block (field) or pot (glasshouse) as random effects.

The number of leaves was statistically analyzed at BBCH 17 for the GH experiment. The elongation of the stem was statistically analyzed at two key moments in the OSR development, at the beginning of the elongation after the wintering period or winter (referred to as stem elongation) and at the beginning of the blooming period (referred to as stem height).

Response variables following a normal distribution (*e.g.*, stem elongation, biomass, yield, and thousand-kernel weight) were log-transformed when necessary. Count data (*e.g.*, plant density, shot-holes, larval counts, oviposition punctures, pollen beetle abundance, branching) were analyzed with GLMMs assuming a negative binomial distribution; models included a zero-inflation component when appropriate. A summary of model specifications and parameters is provided in **Supplementary Table 2**.

Model diagnostics were verified using the DHARMa package (Hartig, 2022). Significance of fixed effects was tested with Wald χ² tests (car package) (Fox and Weisberg, 2019), and significant factors were further explored with pairwise comparisons of estimated marginal means using emmeans (Lenth, 2024), adjusted by the Bonferroni correction (P < 0.05).

#### 2.3.2 Statistical analysis of the glucosinolate concentration

An ANOVA was performed on total GLS content to assess the effects of the experimental setup (GH, Field 2022-2023, Field 2023-2024), sampling campaign (before and after winter), varietal factor, cropping system, and their interaction.

To examine variations in the GLS profile based on experimental conditions and sampling period, a Partial Least Squares Discriminant Analysis (mixOmics package) was applied (Kalivodová et al., 2015; Rohart et al., 2017). Prior to analysis, GLS concentrations were transformed using log(x + 1) to reduce skewness, and then each variable (GLS compound) was range-normalized (min–max rescaling) to the [0,1] interval to ensure comparability across features (Grosjean et al., 2024).

The effects of variety, cropping system, and their interaction were tested separately for each specific GLS and their total concentration variables within each experimental setup (GH, Field 2022-2023, Field 2023-2024) and sampling campaign (before and after wintering or winter). ANOVA (GH experiment) and ANCOVA (field experiments) were used on GLS variables to test the effects of variety, cropping system, and their interaction (with block as a random factor in field trials). When assumptions were violated, data were log+1 transformed; if still unmet, Kruskal–Wallis tests were applied to assess the effects of variety and cropping system.

#### 2.3.3 Statistical analysis of the cropping systems microclimate

In the GH experiment, two sensors per type of cropping system were removed due to dysfunction (n = 40 per cropping system). In the 2022–2023 cropping season, one sensor in monocropping and two sensors in CPS were removed from the analysis (n = 40 for monocrop and n = 32 for intercrop). Due to technical issues in the 2023–2024 cropping season, two sensors for the CPS (n = 16) and three sensors for the monocropping system (n = 24) were kept for the analysis. The average daily temperature was calculated for each sensor. The readings from the control sensors placed outside the canopies were averaged to determine a single daily temperature outside the canopy. For each sensor inside the canopy, the temperature difference was calculated by subtracting the average daily temperature outside the canopy from the temperature recorded by each sensor inside the canopy. For each experiment, a separate linear model was used to evaluate the effect of the cropping system on the difference in temperature under the canopy compared to the external temperature.

The measurements of PAR and R/FR ratio at 5 cm under the canopies and just above the OSR canopy were statistically analyzed. When data sets satisfied ANOVA assumptions, the effect of the cropping system on the response was analyzed with ANOVA; a Kruskal-Wallis test was used to assess the effect of the cropping system on the light parameters.

## 3 Results

### 3.1 Qualitative assessment of the effect of the treatments

We observed contrasting dynamics between oilseed rape (OSR) and faba beans (FB) grown together, depending on the experimental context. In the glasshouse (GH), where plant densities were particularly high, FB rapidly dominated the canopy from early stages. This dominance was further reinforced by the absence of winter frost, preventing natural freezing of the main stems and ultimately leading to a sharp reduction in OSR development, leading to very low seed production, which could not be harvested. Disease incidence was negligible or absent in this controlled setting. In contrast, in the field experiments, despite inter-annual variability, OSR was generally able to overcome interspecific competition and emerge from the FB canopy after winter. This was largely facilitated by the frost during winter, causing the first stems that developed in autumn to freeze, prompting the emergence of new stems in spring, remaining below or at the OSR canopy level.

### 3.2 Morphological traits and crop characteristics of oilseed rape

**Figure 2:**
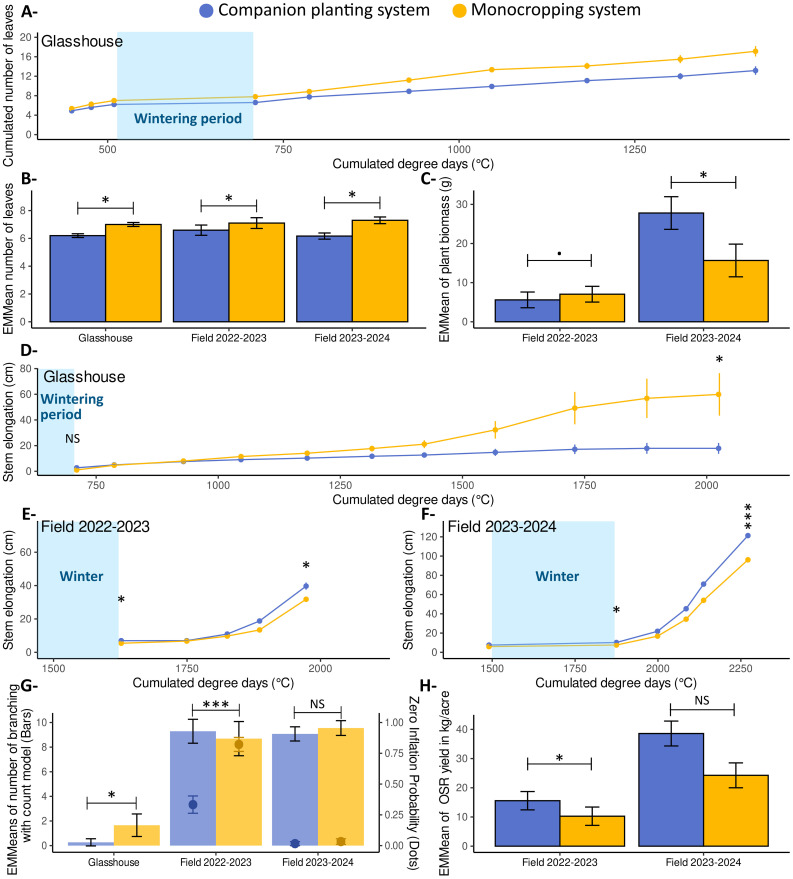
Morphological traits and crop characteristics of the oilseed rape variety Mambo when grown with faba beans or in monocropping. The blue background in the graphs indicates the wintering or winter period in the experiments. **(A)** Cumulated number of leaves during the growing period -Expressed in cumulated degree days- in the glasshouse (GH) experiment. **(B)** Estimated Marginal Means of the number of leaves at BBCH 17 for the GH and 2022–2023 field experiment, and later in the season for the 2023–2024 field experiment. **(C)** Estimated Marginal Means of single plant dry biomass after winter for the field experiments. **(D)** Elongation of the stem in cm after the wintering period to the blooming period in the GH experiment. **(E)** Same measurement for the field experiment in the cropping season 2022-2023. **(F)** Same measurement for the field experiment in the cropping season 2023-2024. **(G)** Predicted counts and zero-inflation probabilities for branching of OSR in companion planting and monocropping systems under the three experimental conditions. The bar plot with the left y-axis represents the predicted count of branching. The zero-inflation probability is shown as dots, with the right y-axis. **(H)** Estimated Marginal Means of OSR yield in kg/acre for the two field experiments. Statistical significance: “.” for p ≤ 0.1, “*” for p ≤ 0.05, “***” for p ≤ 0.001, and “NS” for not significant (p > 0.05), in case of no indication no statistical analysis was applied.

**Figure 3:**
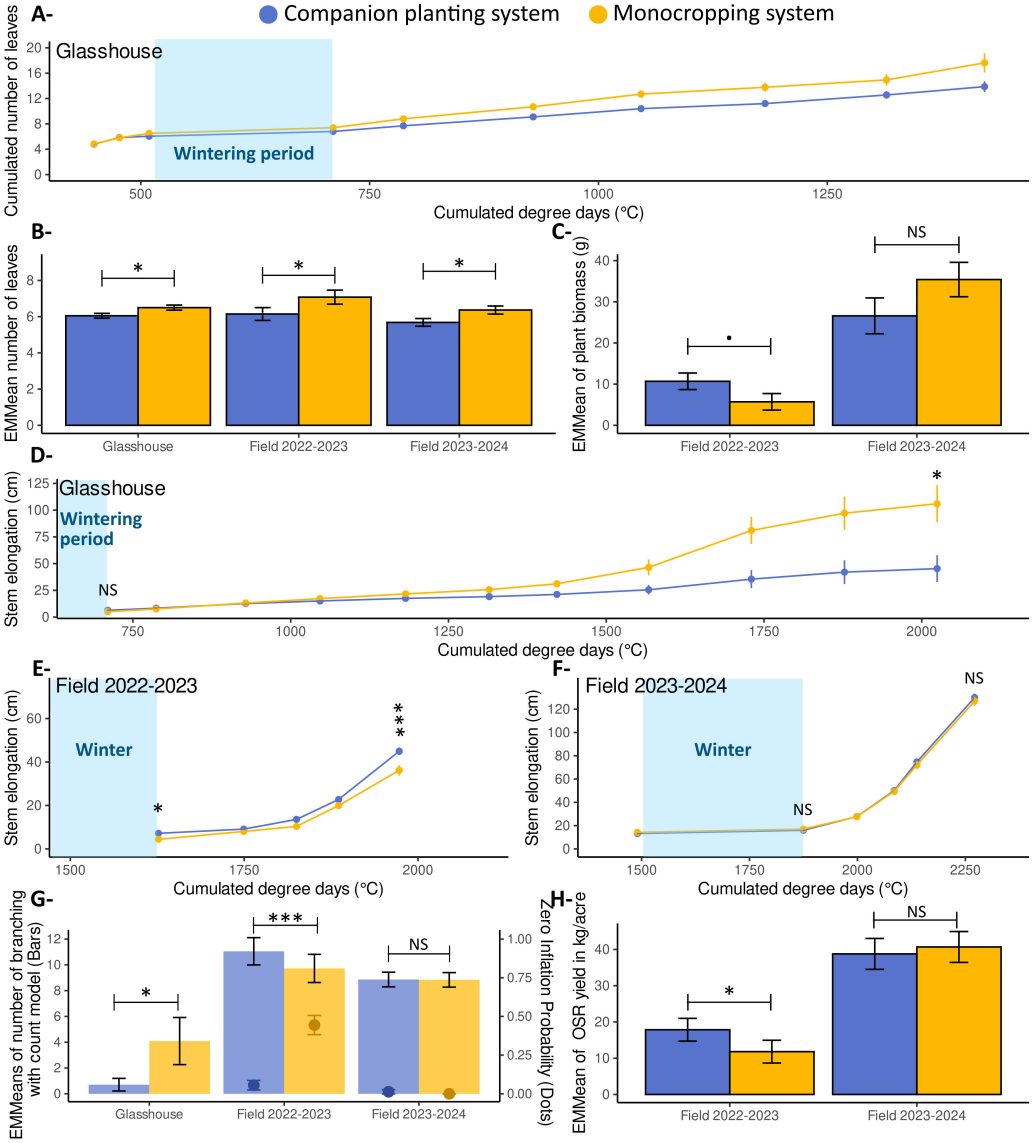
Morphological traits and crop characteristics of the oilseed rape variety Angelico when grown with faba beans or in monocropping. The blue background in the graphs indicates the wintering or winter period in the experiments. **(A)** Cumulated number of leaves during the growing period -Expressed in cumulated degree days- in the glasshouse (GH) experiment. **(B)** Estimated Marginal Means of the number of leaves at BBCH 17 for the GH and 2022–2023 field experiment, and later in the season for the 2023–2024 field experiment. **(C)** Estimated Marginal Means of single plant dry biomass after winter for the field experiments. **(D)** Elongation of the stem in cm after the wintering period to the blooming period in the GH experiment. **(E)** Same measurement for the field experiment in the cropping season 2022-2023. **(F)** Same measurement for the field experiment in the cropping season 2023-2024. **(G)** Predicted counts and zero-inflation probabilities for branching of OSR in companion planting and monocropping systems under the three experimental conditions. The bar plot with the left y-axis represents the predicted count of branching. The zero-inflation probability is shown as dots, with the right y-axis. **(H)** Estimated Marginal Means of OSR yield in kg/acre for the two field experiments. Statistical significance: “.” for p ≤ 0.1, “*” for p ≤ 0.05, “***” for p ≤ 0.001, and “NS” for not significant (p > 0.05), in case of no indication no statistical analysis was applied.

The results of the models for each morphological trait and plant characteristics analyzed in the experiments can be found in **Supplementary Table 3**. The characteristics of the variety Feliciano were very similar to those of Angelico. Therefore, the characteristics of Mambo are shown in **Figure 2**, those of Angelico in **Figure 3**, and those of Feliciano can be found in **Supplementary Figure 1**.

#### 3.2.1 Leaf development

The number of leaves was influenced by the cropping system, with no significant effect of the varietal factor and no interaction between the two factors across the three experiments at the vegetative stage (**Supplementary Table 3**). In the GH, leaf development was slower in OSR grown with companion plants compared to monocropped OSR, with the difference becoming significant once monocropped OSR reached BBCH stage 17 (**Figures 2A**; **3A**, χ² = 4.44, P < 0.05). A similar pattern was observed in the 2022–2023 cropping season, where the number of leaves in OSR grown with companion plants was significantly reduced at BBCH 17 compared to OSR as a sole crop (**Figures 2B**; **3B**, χ² = 4.53, P < 0.05). However, in the 2023–2024 season, no significant difference in the number of leaves was detected at BBCH 17 among treatments (**Supplementary Table 3**). Nevertheless, a significant reduction in the number of leaves in OSR grown with FB compared to monocropped OSR was observed later in the season (**Figure 2B**; **3B**, χ² = 4.87, P < 0.05).

#### 3.2.2 Plant density and biomass

While no significant differences in plant densities were observed among treatments across our two field experiments (**Supplementary Table 3**), the effect on plant biomass varies with the year of experiment. Thus, in the 2022–2023 cropping season, both the varietal factor and the cropping system showed trends toward affecting OSR plant biomass (**Figures 2C**; **3C**, χ² = 4.84, *P* = 0.089; χ² = 3.47, *P* = 0.062, respectively). On the contrary, in the 2023–2024 cropping season, a significant interaction between the varietal factor and the cropping system was observed (χ² = 7.92, *P* < 0.05), with the variety Mambo exhibiting significantly greater plant biomass when grown with FB compared to Mambo monocropped plants (**Figure 2C**, *P* < 0.05).

#### 3.2.3 Stem elongation

Stem elongation was modified in a different way by the cropping systems according to experiments, and the varietal factor had a significant influence on the stem elongation across all experiments (**Supplementary Table 3**). In the GH experiment, the OSR grown with FB tended to have a greater stem elongation than the monocropped OSR just after wintering, but this difference was not significant (**Figures 2D**; **3D**, χ² = 0.34, *P* > 1). In the field, effects varied according to the cropping season. In the 2022–2023 cropping season, the OSR grown with FB had a greater stem elongation than monocropped OSR (**Figures 2E**; **3E**, χ² = 4.86, *P* < 0.05). In the 2023–2024 cropping season, the interaction between the two factors significantly affected stem elongation, with CPS increasing elongation in the variety Mambo but not in the other two varieties (**Figure 2F**, t = 4.976, P < 0.001).

In the GH experiment, the CPS had a significant negative effect on the stem height of the OSR plants (**Figures 2D**; **3D**, χ² = 5.18, *P* < 0.05). In both field experiments, the cropping system effect was in interaction with the varietal effect (**Figures 2E**; **3E**, χ² = 6.64, *P* < 0.05 in 2022–2023 and **Figures 2F**; **3F**, χ² = 13.53, *P* < 0.01 in 2023-2024). In the 2022–2023 cropping season, the variety Mambo and Angelico had significantly higher stems at bloom in CPS than as monocrop (t = 2.921, P < 0.05 and t=4.492, P < 0.001, respectively). In the 2023–2024 cropping season, only the variety Mambo presented a significantly higher stem at bloom when grown with FB compared to monocropped plants (**Figure 2F**, t = 5.607, P < 0.001).

#### 3.2.4 Branching

In the GH experiment, the CPS had a significant negative impact on the number of branches per OSR plants (**Figure 2G**; **3G**, χ² = 4.96, *P* < 0.05). In the first field experiment (2022-2023), the cropping system had a significant influence on the probability of the plant not having any branches (**Figures 2G**; **3G**, χ² = 17.20, *P* < 0.001). The probability for OSR grown with companion plants not to have branches was lower than that of OSR in monocropping in the 2022–2023 cropping season, but no difference was found in the number of branches once the plants had at least one branch (**Supplementary Table 3**). No significant impact of the factors was found in the field trial in 2023-2024.

#### 3.2.5 Thousand kernel weight

No significant difference was found among our treatments in the thousand kernel weight for both field experiments (**Supplementary Table 3**). Yield was strongly correlated with the number of seeds per square meter (**Supplementary Figure 2**, R = 0.98, P < 0.001 for both years).

#### 3.2.6 Yield

In the 2022–2023 cropping season, the CPS plots had a significantly higher OSR yield than those in monocropping (**Figures 2H**; **3H**, χ² = 4.76, P < 0.05). In the 2023–2024 season, the interaction between the varietal factor and the cropping system showed a trend toward an effect on yield (**Figures 2H**; **3H**, χ² = 5.08, P = 0.08). The variety Mambo showed a tendency for higher yield in CPS compared to monocropping (P = 0.163 after Tukey adjustment in pairwise comparison, **Figure 2H**).

#### 3.2.7 Correlation between functional traits and crop performance

**Figure 4:**
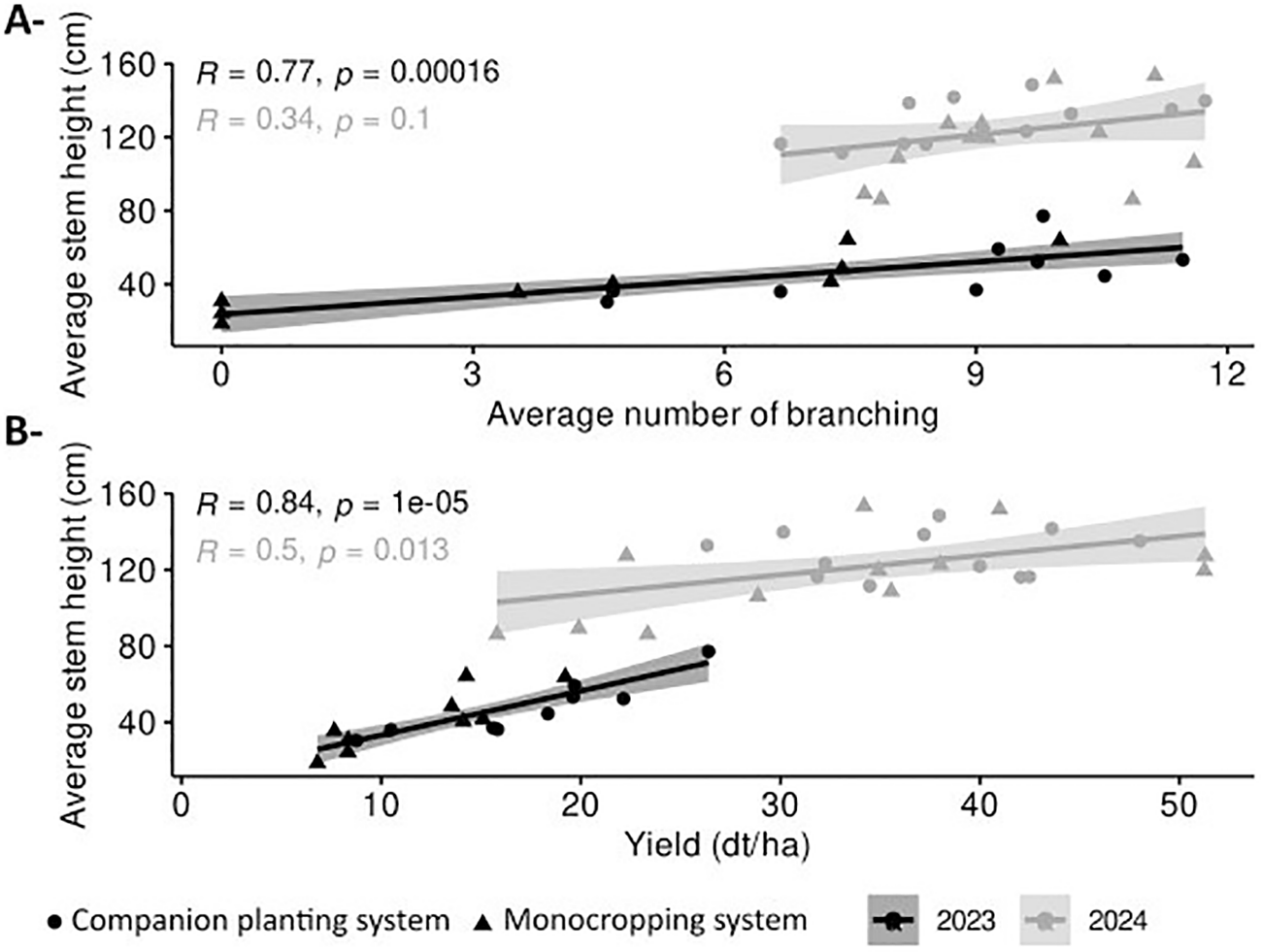
Correlation of the average oilseed rape (OSR) stem height per plot with the **(A)** average number of OSR branching per plot, and **(B)** the OSR yield per plot. A regression line with a confidence interval is shown for each field cropping season. The Pearson correlation coefficient is displayed for each regression (R) with its associated p-value of the t-test (p). Data points’ shapes are determined by the cropping system factor.

The stem height was found to be correlated with the number of branches, which is a yield component, and with the yield, across the two field trials (see **Supplementary Figure 2** for correlation matrix). The correlation with the number of branches was significant in the 2022–2023 field trial and showed a similar trend in the 2023–2024 field trial (**Figure 4A**, R = 0.77, p < 0.01 and R = 0.34, P = 0.1). The correlation of the stem height with the yield was significant in both years (**Figure 4B**, R = 0.84, 0.77, p < 0.001, and R = 0.50, P < 0.5, respectively).

### 3.3 Insect pest incidence

The output of the statistical analysis of OSR insect pest incidence across the two-year field experiments is organized in **Supplementary Table 4**. For both experimental years, no significant effect of the varietal and the cropping system factors was found on the number of shot-holes on cotyledons and on the oviposition number of *C. napi*.

For *P. chrysocephala* larval infestation, no significant effect of the treatments was found on the number of larvae per OSR plants in the 2022–2023 cropping season, but an interaction between our two factors had a significant effect in 2023-2024 (χ² = 6.09, P < 0.05). With the mambo variety having a greater number of larvae per plant than the two other varieties when grown with FB (z = 4.538, p < 0.001 in pairwise comparison with Feliciano and z = 3.490, p < 0.01 in pairwise comparison with Angelico) and the variety Feliciano having fewer larval infestation than the two other varieties when grown in monocrop (z = -3.543, p < 0.01 in pairwise comparison with Mambo and z = -3.410, p < 0.01 in pairwise comparison with Angelico).

For the incidence of *B. aeneus* on inflorescences, the OSR grown with FB had significantly less *B. aeneus* per plant than plants grown as monocrop in the 2022–2023 field experiment (χ² = 8.16, P < 0.01). No impact of the factors on the number of *B. aeneus* on inflorescence was found in the 2023–2024 experimental year.

### 3.4 Metabolic defense traits of oilseed rape – glucosinolates

**Figure 5:**
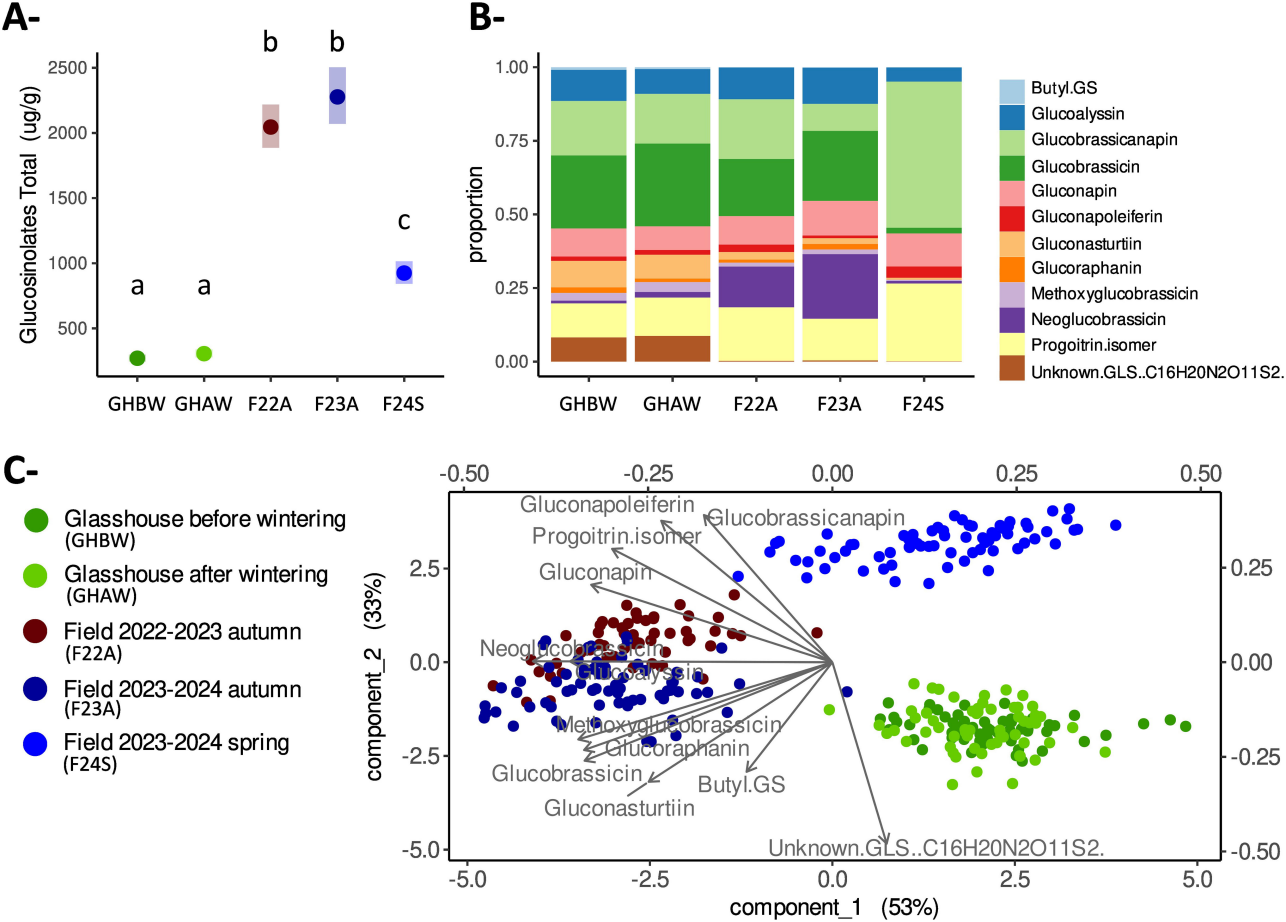
Analysis of the glucosinolates (GLS) concentration in oilseed rape leaves across the experimental conditions and sampling period. **(A)** Estimated Marginal Means of the concentration of total GLS (µg.g-1) in leaves for each experimental condition and sampling periods. Letters above the graphic show group of significance with p < 0.05. **(B)** Proportions of each specific GLS for each experimental condition and sampling period. **(C)** Score plot combined with loading plots of the Partial Least Squares—Discriminant Analysis performed on the standardized concentration of each of the GLS types. Scores are represented on the bottom and left axes, whereas variable loadings (arrows) are represented on the top and right axes.

Leaf GLS concentration was significantly affected by the sampling period and experimental conditions (**Figure 5A**, F = 506.2, P < 0.001) but not by the cropping system or varietal factor in the same model. Leaf samples from the GH experiment, both before and after wintering (271.12 ± 13.86 µg.g^-1^ and 306.26 ± 15.77 µg.g^-1^, respectively), contained seven times less GLS than field samples in autumn (t = 28, P < 0.001 for all GH - field in autumn comparisons). Field samples from autumn had the highest GLS concentrations with 2045 ± 84.47 µg.g^-1^ in 2022–2023 and 2276.48 ± 110.34 µg.g^-1^ in 2023–2024 cropping seasons. Spring field samples (924.83 ± 44.10 µg.g^-1^) also had significantly lower GLS levels than autumn field samples (t = -12.58, P < 0.001 for 2022–2023; t = -15.26, P < 0.001).

Given the observed differences in total GLS content, we further examined individual GLS to determine whether these changes reflected a general response across all compounds or shifts in specific components of the GLS profile. **Figure 5B** shows distinct differences in specific GLS proportions between the experimental conditions of GH, of the field in autumn, and of the field in spring. In the GH experimental conditions, unknown GLS and gluconasturtiin were over-expressed, while neoglucobrassicin and progointrin were under-expressed compared to field conditions in autumn. The sampling period in the field experiment affected the leaf GLS profile. In spring, progoitrin and glucobrassicanapin were present in high quantities, while glucobrassicin and neoglucobrassicin proportions were much lower than in autumn field samples.

The PLS-DA analysis discriminated three groups according to the proportion of specific GLS in the samples (**Figure 5C**). The first group includes GH experiment samples, where the sampling period (before or after wintering) did not alter the profile enough to be distinct. The second group consists of autumn field samples from both years, which are similar and not fully distinguishable from one another, even if not completely overlapping. The third group contains only spring samples from the 2023–2024 field experiment and clearly distinguishes itself from the samples of the GH experiment and the fields in autumn.

**Figure 6:**
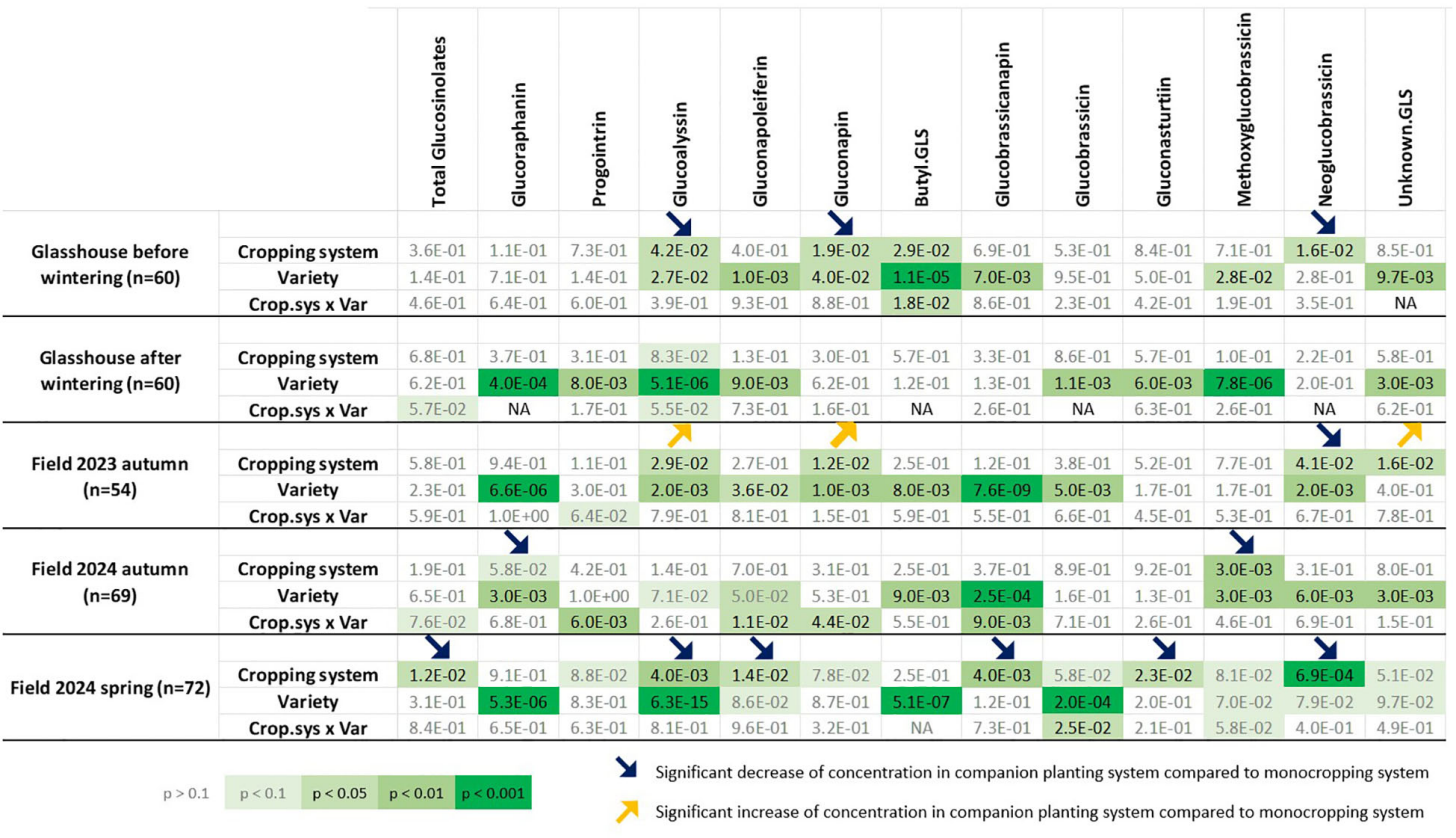
Effect of oilseed rape (OSR) companion planting with faba beans as compared to monocrop OSR and OSR varietal factors, and their interaction on the concentration of specific glucosinolates (GLS) across experimental conditions and sampling periods. The P-values from ANOVA for each GLS across experimental conditions and sampling periods are displayed for each factor. Levels of significance are highlighted with a green hue, from lighter green (p<0.1) to darker green (p<0.001). Arrows show the effect of companion planting on OSR-specific GLS compared to monocropped ones. Blue arrows pointing downward show a reduction of the GLS concentration in companion planting compared to monocropping. The yellow arrow pointing upward shows an increase in the GLS concentration in companion planting compared to monocropping. The arrows are displayed when the cropping system factor significantly (p < 0.05) impacts the GLS concentration without interaction with the varietal factor.

The experimental conditions and sampling period having a strong impact on the total concentration of GLS and their profiles, analysis for the effect of the treatments were performed on each sampling campaign independently (**Figure 6**). The treatments had a significant impact on the total GLS content only for the samples from the field 2023–2024 experiment sampled in spring (F = 6.70, P < 0.05 for the cropping system). The OSR plants grown with FB in the 2023–2024 spring had an estimated marginal means of total GLS of 848.84 ± 56.70 µg.g^-1^, significantly reduced from that of monocropped plants, estimated to 1083.90 ± 72.39 µg.g^-1^. Most specific GLS were reduced in CPS for this sampling campaign in the field compared to monocropping.

Varietal factor had a significant impact on the concentration of the majority of the specific GLS in the GH samples and the autumn field samples. The cropping system moderately impacted the concentration of specific GLS in the GH and the autumn field samples; CPS mostly decreased their concentration, except for the glucoalyssin and the gluconapin in the samples from autumn 2022-2023. In a few cases, the interaction of the varietal and the cropping system factors significantly impacted the concentration of specific GLS. Beyond the effect per experimental campaign, certain effects can be observed over several campaigns for certain GLS. This is particularly the case for the neoglucobrassicin that was found to be decreased by CPS in three sampling campaigns out of five.

### 3.5 Microclimate characterization

The OSR-FB CPS had a strong impact on the crop microclimate in both GH and field experimental conditions (**Supplementary Table 5**).

**Figure 7:**
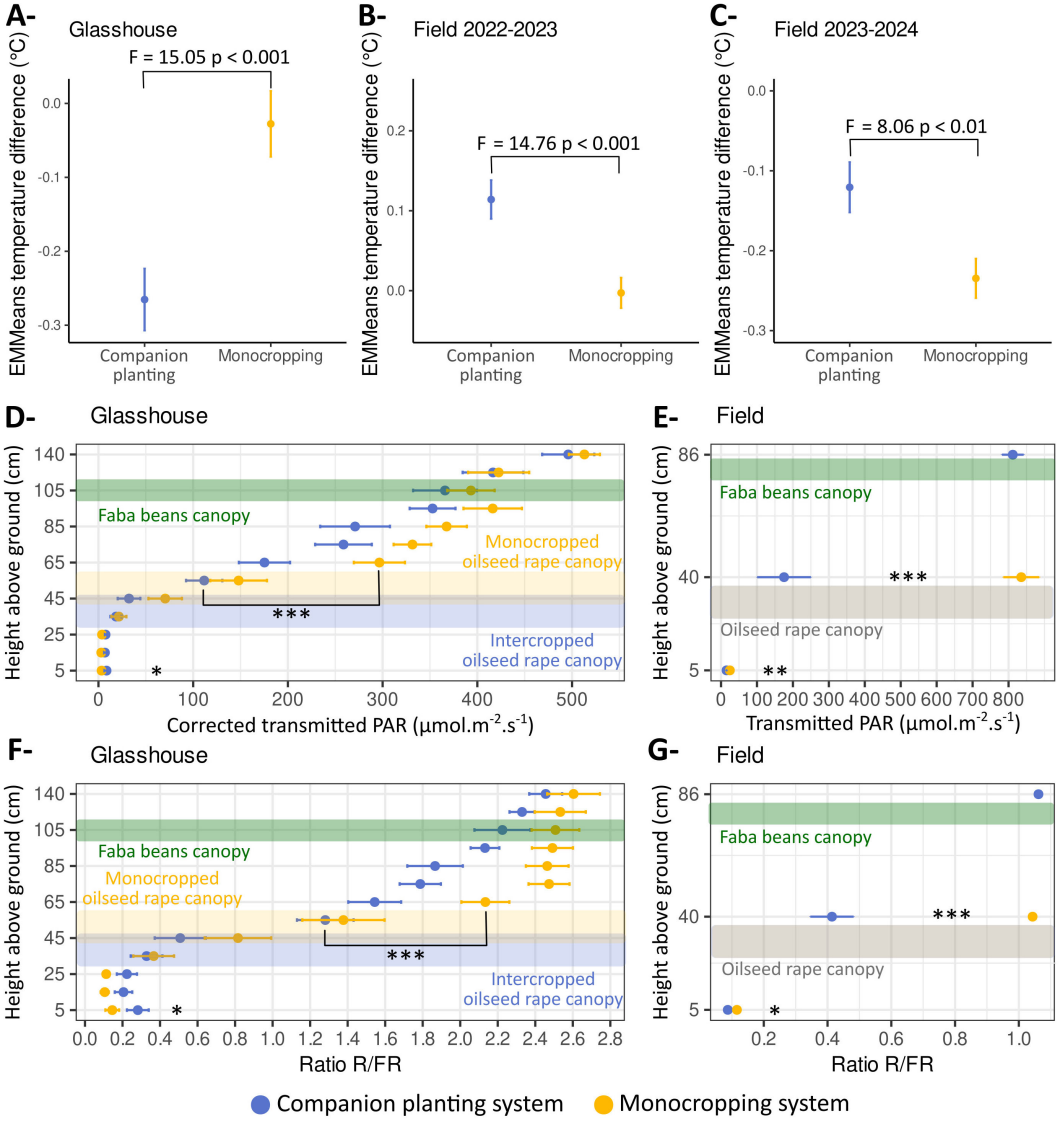
Characteristics of the oilseed rape (OSR) canopy microclimate in relation to the cropping system under glasshouse (GH) and field conditions. The companion planting treatment consisted of an additive companion planting of OSR with faba beans (FB). **(A)** Estimated Marginal Means of the temperature difference between the outside environment and the area beneath the OSR canopies, depending on the cropping system for the GH experiment. **(B)** Same measurement for the field experiment in the cropping season 2022-2023. **(C)** Same measurement for the field experiment in the cropping season 2023-2024. **(D)** Corrected photosynthetic activity radiation (PAR) along the canopy of the two cropping systems in GH conditions. **(E)** Corrected PAR on the ground under canopies, above the OSR canopy, and above the FB canopy in field conditions for the two cropping systems (2023-2024). **(F)** Red on far red ratio (R/FR) along the canopy of the two cropping systems in GH conditions. **(G)** R/FR on the ground under canopies, above the OSR canopy, and above the FB canopy in field conditions for the two cropping systems (2023-2024). Statistical significance: “.” for p ≤ 0.1, “*” for p ≤ 0.05, “***” for p ≤ 0.001, and “NS” for not significant (p > 0.05), in case of no indication no statistical analysis was applied.

The effect of the cropping system on the air temperature under the canopy had a differing effect depending on the experimental conditions. The temperature under the canopy was significantly reduced by CPS in the GH experiment (**Figure 7A**, F = 15.05, P < 0.001). However, the temperatures under the canopies of the CPS in the field experiments were significantly increased compared to monocrop (**Figures 7B, C**, F = 14.76, P < 0.001, and F = 8.06, P < 0.01, respectively).

The light quality and quantity in the GH depend on the distance to the lamps. This explains the steady increase in PAR and R/FR ratio above the OSR canopy, even without FB (**Figures 7D, F**). In contrast, this effect is absent in the field with natural sunlight (**Figures 7E, G**). Moreover, the R/FR ratio under the greenhouse lamps was 2.5, which is notably different from the natural light ratio, typically around 1, observed in the field experiment. Under GH conditions, the canopy of OSR grown with FB was lower than that of monocropped OSR (**Figures 7D, E**), whereas in the field, the canopies reached similar heights. Therefore, for the analysis of light conditions above the OSR canopy in the GH, measurements were taken at 55 cm from the ground for the CPS and at 65 cm for the monocropped system. In contrast, under field conditions, light measurements above the canopy were taken at 40 cm from the ground for both treatments.

Photosynthetically active radiation at the soil surface under both cropping systems’ canopies was very low in both GH and field experiments. However, in the GH experiment, the monocropped canopy intercepted more light than the CPS one (**Figure 7D**, χ² = 4.85, P < 0.05), with an incident PAR at 5cm above the ground of 3.31 ± 0.64 µmol.m-2.s-1 for the monocrop system and 8.52 ± 3.12 µmol.m-2.s-1 for CPS. Conversely, in the field experiment, the canopy of OSR grown with companion plants intercepted more light than the monocropped canopy (**Figure 7E**, F = 9.06, *P* < 0.01), with an incident PAR at 5 cm above the ground of 24.62 ± 1.96 µmol.m-2.s-1 for the monocrop system and 15.30 ± 2.40µmol.m-2.s-1 for CPS. Just above the OSR canopy (55–65 cm in GH, 40 cm in the field), the PAR was significantly impacted by the cropping system in both experiments (**Figures 7F, G**, χ² = 16.60, P < 0.001 in GH; χ² = 10.965, P < 0.001 in the field). The PAR above the OSR canopy in the CPS was 111.63 ± 19.14 µmol.m-2.s-1 in GH and 175.38 ± 74.84 µmol.m-2.s-1 in the field, both lower than in monocropping (296.61 ± 26.98 µmol.m-2.s-1 in GH, 835.42 ± 49.84 µmol.m-2.s-1 in the field), traducing the shading effect of CPS on OSR.

The R/FR ratio was significantly lower under the monocropped canopy than the canopy of CPS in GH (**Figure 7F**, χ² = 6.43, P < 0.05), with an R/FR ratio of 0.14 ± 0.04 at 5 cm above ground under the monocrop canopy and 0.28 ± 0.06 under the canopy of CPS. The inverse was found in the field, with R/FR ratio under canopy of CPS (0.09 ± 0.01) significantly lower than the one under monocropped canopy (0.12 ± 0.01), even though values are very low in both situations (**Figure 7G** and F = 5.39, P < 0.05). Above the OSR canopy, the R/FR ratio was lower in CPS than in monocropping in both experiments (**Figures 7F, G**, χ² =14.00, P < 0.001 in GH; χ² = 12.79, P < 0.001 in the field). Just above the OSR canopy (55–65 cm in GH, 40 cm in the field), the R/FR ratio in CPS was 1.28 ± 0.15 in GH and 0.41 ± 0.07 in the field, both lower than in monocropping (2.13 ± 0.13 in GH, 1.04 ± 0.005 in the field). The low R/FR value of 0.41 observed in the field indicates that OSR grown with FB experienced a shade avoidance trigger near the top of the canopy, unlike monocropped OSR. In contrast, under glasshouse conditions, the R/FR ratio decreased below 1 only in the lower layers of the CPS canopy.

## 4 Discussion

This study provides new insights into the mechanisms underlying the performance of oilseed rape (OSR*, Brassica napus*) in companion planting systems (CPS) with faba bean (FB, *Vicia faba*) by integrating ecophysiological, chemical ecology, and agronomic approaches across different OSR genetic backgrounds. While companion plants are commonly studied for their contribution to soil fertility, weed suppression, and pest regulation, our results demonstrate that they can also directly contribute to crop performance by modifying OSR aboveground functional traits. We showed that FB companion planting induced contrasting responses in OSR, with early competition during vegetative growth followed by compensatory mechanisms during reproductive development, allowing yield maintenance or improvement under non-limiting nitrogen field conditions. In parallel, glucosinolate-based defenses were primarily shaped by environmental conditions and varietal identity, highlighting the importance of considering both crop genetic background and plant plasticity when designing CPS.

According to previously published studies, we expected lower pest incidence in OSR grown with FB compared to monoculture (Breitenmoser et al., 2022; Coston et al., 2022; Emery et al., 2021; Magnin et al., 2025; Ruck et al., 2018; Seimandi-Corda et al., 2024). However, in our two-year field experiments, pest incidence in CPS plots was generally low and similar to that of monocropped plots for most monitored pests. This could be due to the overall field setup, where the monitored plots were part of a larger field of OSR grown with companion plants. As a result, the potential protective effects of CPS (such as visual and chemical disruption) may have influenced all plots, including the monocropped ones (Magnin et al., 2025; Seimandi-Corda et al., 2024). These pest pressure conditions allowed us to compare the two cropping systems in terms of their functional traits and yield, without the confounding influence of pest damage.

### 4.1 Faba bean competition reduces oilseed rape vegetative development mainly through light limitation

Oilseed rape plants grown with FB consistently showed a reduction in the number of leaves compared with monocropped plants across experimental conditions. This indicates that the presence of FB during the vegetative phase altered OSR development, even under non-limiting nitrogen conditions. Similar reductions in leaf appearance have been reported under competitive environments and are generally associated with changes in resource availability and plant perception of neighboring vegetation (Ballaré et al., 1987; Casal, 2013).

Among the environmental factors modified by the presence of FB, temperature appeared unlikely to be the main driver of the observed developmental delay. Indeed, although leaf appearance rate is strongly regulated by accumulated degree days (Habekotté, 1997), the contrasting effects of CPS on canopy temperature between GH and field conditions did not match the consistent reduction observed in OSR development. This suggests that changes in accumulated degree days were not sufficient to explain the response of OSR plants to CPS.

In contrast, changes in the light environment provide a more consistent explanation for the developmental delay observed in CPS. In both GH and field conditions, FB developed a taller canopy than OSR during the vegetative phase, reducing the amount of photosynthetically active radiation reaching OSR plants. Although light quality (R/FR ratio) differed between GH and field conditions, the consistent reduction in light quantity experienced by OSR plants in CPS suggests that shading was the dominant factor affecting vegetative development.

Overall, these results indicate that the dominance of the FB canopy during the early growth stages induced a competitive effect on OSR mainly through light limitation. Since the number of leaves at the vegetative stage determines the branching potential of the plant (Mendham and Scott, 1975), OSR plants grown with FB entered the reproductive phase with a lower initial yield potential compared to monocropped plants.

### 4.2 Dynamic interactions between faba bean competition and oilseed rape reproductive development determine yield responses

The contrasting effects of CPS observed between GH and field conditions in reproductive development highlight the importance of the timing and duration of competition between FB and OSR. Under GH conditions, the continuous growth of FB maintained strong light competition throughout both vegetative and reproductive stages, preventing OSR plants from completing their reproductive development. This response was likely driven by prolonged shading (reduced light quantity) rather than by light quality alone, as the artificial light conditions generated R/FR ratios that differed from those usually observed under natural canopy competition.

In contrast, under field conditions, frost damage of FB resulted in a temporal shift from early competition to its subsequent release. During autumn, the dominance of the FB canopy modified the light environment experienced by OSR plants, potentially triggering shade-avoidance responses. A reduced R/FR ratio is known to promote resource allocation toward stem elongation as a strategy to escape competition (Franklin, 2008). This suggests a potential “priming effect” of light quality, where the R/FR ratio conditions the OSR plant resource allocation strategy, enhancing its capacity for elongation when light competition is reduced.

These morphological adjustments likely contributed to maintaining or improving yield performance in CPS. The number of grains per unit area was closely associated with plant architectural traits, particularly stem elongation and branching, which is consistent with previous studies identifying these traits as key determinants of OSR yield formation in genetic studies (Diepenbrock, 2000; Saroj et al., 2021). Therefore, the higher yield observed in CPS under certain conditions may result from improved reproductive architecture following the release from early interspecific competition rather than from a direct facilitative effect of FB during the entire crop cycle. As a result, the yield increase observed in the CPS during our field experiment is better understood as an enhanced capacity of unlocking potential in the reproductive phase compared to OSR as a monocrop rather than an increase in yield potential.

Additional factors may also have contributed to yield responses in CPS. The reduction of *B. aeneus* incidence observed in the presence of FB confirms previous findings showing that companion plants can contribute to pest regulation in OSR systems (Breitenmoser et al., 2022; Magnin et al., 2025). Similarly, improved biomass accumulation in some CPS treatments may have favored plant performance but could also explain higher *P. chrysocephala* larval infestation, as infestation levels have previously been associated with plant biomass (Seimandi-Corda et al., 2024). However, given the relatively low pest pressure observed, this effect was probably not the main driver of yield differences.

The spatial arrangement of plants may influence the effects of CPS on OSR traits. In particular, differences in row spacing and in the spatial arrangement of FB (random undersowing versus inter-row sowing) between the two field experiments may partly explain the contrasting effects of CPS on OSR biomass observed between years. These results suggest that the performance of OSR–FB systems depends not only on species interactions, but also on their spatial configuration. Further research is therefore needed to identify the optimal arrangement of companion plants to maximize agronomic benefits while limiting competition.

### 4.3 Plasticity of oilseed rape glucosinolate profiles across environments, genotypes, and cropping systems

The experimental growing conditions significantly influenced the leaf glucosinolate (GLS) content in OSR, with notable effects on both total GLS content and specific GLS profiles, which aligned with previous studies on other *Brassica* species (Oloyede et al., 2021). The total GLS concentration in the leaves was approximately seven times higher in field-grown OSR compared to plants cultivated in the GH, whatever the period considered (pre- or post-winter). Additionally, the composition of GLS differed between the two environments. Glasshouse-grown OSR had a higher proportion of unknown GLS, glucoraphanin, and butyl-GLS in their leaves, while field-grown OSR contained a higher proportion of neoglucobrassicin, consistent with observations made by Sarwar and Kirkegaard (1998) on spring OSR cultivars.

Interestingly, the autumn samples from the two field experiments showed similar total GLS concentrations and GLS profiles, suggesting that year-to-year variations in environmental conditions in the open field may not be sufficient to significantly affect the GLS content of OSR. In contrast, GH conditions appear to create drastic changes in GLS content due to the controlled environment. Several factors differ between field and GH conditions, including soil substrate, plant nutrition, light quality, temperature, and biotic factors such as pest pressure. We suggest that further research is needed to fully understand the implications of these factors before screening GLS in varieties or applying other treatments in GH conditions, as the results may not be directly translatable to field conditions.

The natural winter conditions in the field markedly affected both the total concentration and profile of GLS. Thus, the timing of sampling under field conditions was particularly influential. More specifically, GLS content was approximately twice as high in samples collected before winter compared to those collected after winter. Additionally, post-winter samples exhibited an overexpression of glucobrassicanapin, while indolyl GLS and glucoraphanin, which were present before winter, were nearly undetectable after winter. This suggests that winter conditions not only reduced the total GLS content but also triggered changes in GLS biosynthesis pathways, favoring certain compounds over others. These findings highlight the dynamic and environment-dependent nature of GLS production in OSR (Engelen-Eigles et al., 2006; Justen, 2010; Park et al., 2019; Sarwar and Kirkegaard, 1998).

The relative impact of variety and cropping system factors on GLS is context-dependent, as their effects vary between controlled GH and open-field conditions, across different years, and over time within the same setting. The treatments had a significant influence on the total concentration of GLS, uniquely for samples from the field experiment after winter. The OSR plants grown with FB had a reduction in their leaf total GLS as compared to plants grown as a monocrop in spring 2024. This finding suggests that CPS had a stronger impact on glucosinolate synthesis and accumulation during winter, likely due to altered environmental stress responses or resource allocation.

The glucosinolate profiles were significantly influenced by both the cropping system and the variety, but the effect of the treatments depended on the experimental conditions. In both the GH and the vegetative stage of the field experiment, the varietal factor had a stronger impact on specific GLS concentrations than the cropping system, suggesting that genetic differences among varieties play a more critical role at this early growth phase and in low disturbance conditions such as those in GH. However, we observed that when the cropping system significantly affected specific GLS concentrations, CPS reduced the concentration of a particular GLS in eleven cases out of fourteen. Thus, CPS modulates the production of glucosinolate defense compounds, which may reduce OSR attractiveness to insect pests. All exceptions occurred in samples from the 2022–2023 autumn field samples, highlighting the interactions between environmental conditions and treatment. Further investigations are needed to better understand the production of glucosinolates and their subsequent effect on pests.

## Data Availability

The datasets presented in this study can be found in online repositories. The names of the repository/repositories and accession number(s) can be found below: https://github.com/LaurieMagnin/Functional-Traits-OSR-FB.
