# Supplementary material for: Unravelling trait-mediated effects of genotype and companion planting system on oilseed rape performance
Source: Front Plant Sci. 2026 Jul 30;17:1878300. doi: 10.3389/fpls.2026.1878300 (PMC13470748; doi:10.3389/fpls.2026.1878300)
Supplement: Supplementary file 1 [file SupplementaryFile1.docx]

Supplementary Material

# 1 Supplementary Figures and Tables

**Box. 1.** Oilseed rape growing conditions in glasshouse experiment

During the first month of growth, plants were maintained under a photoperiod of 14 hours with supplemented lighting, providing a minimum of photosynthetic photon flux density (PPFD) of 330 µmol.m⁻².s⁻¹. Daytime and nighttime temperatures were controlled at 17°C and 9°C, respectively. Forty days after sowing, corresponding to 534 cumulative degree days (in 0°C base) after OSR emergence, the containers were transferred to a cold chamber for 28 days of vernalisation. The chamber was illuminated with a PPFD of 200 µmol.m⁻².s⁻¹ for 6 hours per day. The temperature was maintained at 4°C for the first 15 days, lowered to 0°C for the subsequent 7 days, and then returned to 4°C for the final 6 days of the vernalisation period. The containers were set back in the glasshouse with 14 hours lighting supplementation providing a minimum PPFD of 330 µmol.m^-2^.s^-1^. Daytime and nighttime temperatures were controlled at 20°C and 15°C, respectively.

**Table S1.** Replication statement.

| **Scale of inference** | **Response** | **Experiment** | **Factor** | **Scale at which the factor of interest was applied** | **Number of replicates at the appropriate scale** | **Random factor** |
| --- | --- | --- | --- | --- | --- | --- |
| Plant physiological traits | Leaf development at BBCH 17 | Glasshouse | Cropping sys. And Variety | plant | n = 20 per treatment | Pot |
|  |  | Field 2022-2023 | Cropping sys. And Variety | plant | n = 45 per treatment | Block |
|  |  | Field 2023-2024 | Cropping sys. And Variety | plant | n = 60 per treatment | Block |
|  | Biomass | Field 2022-2023 | Cropping sys. And Variety | five pooled plants | n = 3 per treatment | Block |
|  |  | Field 2023-2024 | Cropping sys. And Variety | three pooled plants | n = 12 per treatment | Block |
|  | Stem elongation | Glasshouse | Cropping sys. And Variety | plant | n = 20 per treatment | Pot |
|  |  | Field 2022-2023 | Cropping sys. And Variety | plant | n = 45 per treatment | Block |
|  |  | Field 2023-2024 | Cropping sys. And Variety | plant | n = 60 per treatment | Block |
|  | Stem height | Glasshouse | Cropping sys. And Variety | plant | n = 20 per treatment | Pot |
|  |  | Field 2022-2023 | Cropping sys. And Variety | plant | n = 45 per treatment | Block |
|  |  | Field 2023-2024 | Cropping sys. And Variety | plant | n = 60 per treatment | Block |
|  | Branching | Glasshouse | Cropping sys. And Variety | plant | n = 12 per treatment | Pot |
|  |  | Field 2022-2023 | Cropping sys. And Variety | plant | n = 45 per treatment | Block |
|  |  | Field 2023-2024 | Cropping sys. And Variety | plant | n = 60 per treatment | Block |
|  | Glucosinolates before wintering or winter | Glasshouse | Cropping sys. And Variety | plant | n = 10 per treatment | Pot |
|  |  | Field 2022-2023 | Cropping sys. And Variety | three pooled plants | n = 9 per treatment | Block |
|  |  | Field 2023-2024 | Cropping sys. And Variety | three pooled plants | n = 12 per treatment | Block |
|  | Glucosinolates after wintering or winter | Glasshouse | Cropping sys. And Variety | plant | n = 10 per treatment | Pot |
|  |  | Field 2023-2024 | Cropping sys. And Variety | three pooled plants | n = 12 per treatment | Block |
| Crop characteristics | Plants density | Field 2022-2023 | Cropping sys. And Variety | plot | n = 3 per treatment | Block |
|  |  | Field 2023-2024 | Cropping sys. And Variety | plot | n = 4 per treatment | Block |
|  | Thousand kernel weight | Field 2022-2023 | Cropping sys. And Variety | plot | n = 3 per treatment | Block |
|  |  | Field 2023-2024 | Cropping sys. And Variety | plot | n = 4 per treatment | Block |
|  | Yield | Field 2022-2023 | Cropping sys. And Variety | plot | n = 3 per treatment | Block |
|  |  | Field 2023-2024 | Cropping sys. And Variety | plot | n = 4 per treatment | Block |
| Insect pest incidence at the plant scale | Shotholes on cotyledons | Field 2022-2023 | Cropping sys. And Variety | plant | n = 30 per treatment | Block |
|  |  | Field 2023-2024 | Cropping sys. And Variety | plant | n = 60 per treatment | Block |
|  | *Psylloides chrysocephala* larval infestation | Field 2022-2023 | Cropping sys. And Variety | five pooled plants | n = 3 per treatment | Block |
|  |  | Field 2023-2024 | Cropping sys. And Variety | three pooled plants | n = 12 per treatment | Block |
|  | *Ceuthorynchus napi* oviposition on stems | Field 2022-2023 | Cropping sys. And Variety | plant | n = 45 per treatment | Block |
|  |  | Field 2023-2024 | Cropping sys. And Variety | plant | n = 60 per treatment | Block |
|  | *Brassicogethes aeneus* on inflorescence | Field 2022-2023 | Cropping sys. And Variety | plant | n = 45 per treatment | Block |
|  |  | Field 2023-2024 | Cropping sys. And Variety | plant | n = 60 per treatment | Block |
| Microclimatic characteristics of the crop | Temperature under canopy | Glasshouse | Cropping sys. | pot | n = 10 per treatment | side of the glasshouse |
|  |  | Field 2022-2023 | Cropping sys. | plot | n = 6 per treatment | Block |
|  |  | Field 2023-2024 | Cropping sys. | plot | n = 6 per treatment | Block |
|  | PAR under canopy | Glasshouse | Cropping sys. | pot | n = 14 per treatment | side of the glasshouse |
|  |  | Field | Cropping sys. | plot | n = 9 per treatment | Block |
|  | PAR above OSR canopy | Glasshouse | Cropping sys. | pot | n = 14 per treatment | Pot |
|  |  | Field | Cropping sys. | plot | n = 9 per treatment | Block |
|  | R:FR under canopy | Glasshouse | Cropping sys. | pot | n = 14 per treatment | Pot |
|  |  | Field | Cropping sys. | plot | n = 9 per treatment | Block |
|  | R:FR above OSR canopy | Glasshouse | Cropping sys. | pot | n = 14 per treatment | Pot |
|  |  | Field | Cropping sys. | plot | n = 9 per treatment | Block |

**Table S2.** Summary table of the statistical models applied on the responses in the glasshouse and two-years field experiments and their parameters.

| **Response** | **Experiment** | **Model** | **Link** | **Distribution** | **Random factor** |
| --- | --- | --- | --- | --- | --- |
| Leaf development at BBCH 17 | Glasshouse | GLMMs | log | Compois | Pots |
|  | Field 2022-2023 | GLMMs | log | Compois | Block |
|  | Field 2023-2024 | GLMMs | log | Compois | Block |
| Biomass | Field 2022-2023 | Lmer | - | - | Block |
|  | Field 2023-2024 | Lmer | - | - | Block |
| Plants density | Field 2022-2023 | GLMMs | log | Negative binomial | Block |
|  | Field 2023-2024 | GLMMs | log | Negative binomial | Block |
| Stem elongation | Glasshouse | Lmer | log transformation | | Pots |
|  | Field 2022-2023 | Lmer | log transformation | | Block |
|  | Field 2023-2024 | Lmer |  | | Block |
| Stem height | Glasshouse | Lmer | log transformation | | Pots |
|  | Field 2022-2023 | Lmer | log transformation | | Block |
|  | Field 2023-2024 | Lmer | log transformation | | Block |
| Branching | Field 2022-2023 | Zero inflated GLMMs | log | Negative binomial | Block |
|  | Field 2023-2024 | Zero inflated GLMMs | log | Negative binomial | Block |
| Thousand kernel weight | Field 2022-2023 | Lmer | - | - | Block |
|  | Field 2023-2024 | Lmer | - | - | Block |
| Seed density | Field 2022-2023 | Lmer | - | - | Block |
|  | Field 2023-2024 | Lmer | - | - | Block |
| Yield | Field 2022-2023 | Lmer | - | - | Block |
|  | Field 2023-2024 | Lmer | - | - | Block |
| Shotholes on cotyledons | Field 2022-2023 | GLMMs | log | Negative binomial | Block |
|  | Field 2023-2024 | GLMMs | log | Negative binomial | Block |
| *Psylliodes chrysocephala* larval infestation | Field 2022-2023 | GLMMs | log | Negative binomial | Block |
|  | Field 2023-2024 | GLMMs | log | Negative binomial | Block |
| *Ceutorhynchus napi* oviposition on stems | Field 2022-2023 | Zero inflated GLMMs | log | Negative binomial | Block |
|  | Field 2023-2024 | Zero inflated GLMMs | log | Negative binomial | Block |
| *Brassicogethes aeneus* on inflorescence | Field 2022-2023 | GLMMs | log | Negative binomial | Block |
|  | Field 2023-2024 | Zero inflated GLMMs | log | Negative binomial | Block |

**Table S3.** Summary table showing the output of statistical models applied to oilseed rape morphological traits and yield components, analyzing the effects of two factors (variety and cropping system) and their interaction across the different experimental campaigns.

| **Response** | **Experiment** | **Factor** | **Chisq** | **Df** | **P-value** |  |
| --- | --- | --- | --- | --- | --- | --- |
| Leaf development at BBCH 17 | Glasshouse | Variety | 0.54 | 2 | 7.64E-01 |  |
|  |  | **Cropping sys.** | **4.44** | **1** | **3.50E-02** | ***** |
|  |  | Variety:Cropping sys. | 1.27 | 2 | 5.29E-01 |  |
|  | Field 2022-2023 | Variety | 1.13 | 2 | 5.69E-01 |  |
|  |  | **Cropping sys.** | **4.53** | **1** | **3.33E-02** | * |
|  |  | Variety:Cropping sys. | 0.58 | 2 | 7.50E-01 |  |
|  | Field 2023-2024 | Variety | 2.93 | 2 | 2.31E-01 |  |
|  |  | Cropping sys. | 0.00 | 1 | 9.47E-01 |  |
|  |  | Variety:Cropping sys. | 1.29 | 2 | 5.24E-01 |  |
| Leaf development in november | Field 2023-2024 | Variety | 2.72 | 2 | 2.57E-01 |  |
|  |  | **Cropping sys.** | **4.87** | **1** | **2.74E-02** | * |
|  |  | Variety:Cropping sys. | 0.70 | 2 | 7.04E-01 |  |
| Biomass | Field 2022-2023 | **Variety** | **4.84** | **2** | **8.90E-02** | . |
|  |  | **Cropping sys.** | **3.47** | **1** | **6.23E-02** | . |
|  |  | Variety:Cropping sys. | 4.08 | 2 | 1.30E-01 |  |
|  | Field 2023-2024 | Variety | 0.46 | 2 | 7.96E-01 |  |
|  |  | Cropping sys. | 2.33 | 1 | 1.27E-01 |  |
|  |  | **Variety:Cropping sys.** | **7.92** | **2** | **1.91E-02** | * |
| Plants density | Field 2022-2023 | Variety | 4.48 | 2 | 1.06E-01 |  |
|  |  | Cropping sys. | 1.28 | 1 | 2.58E-01 |  |
|  |  | Variety:Cropping sys. | 3.06 | 2 | 2.16E-01 |  |
|  | Field 2023-2024 | Variety | 3.83 | 2 | 1.48E-01 |  |
|  |  | Cropping sys. | 0.54 | 1 | 4.62E-01 |  |
|  |  | Variety:Cropping sys. | 0.15 | 2 | 9.28E-01 |  |
| Stem elongation | Glasshouse | Variety | **24.73** | **2** | **4.27E-06** | ******* |
|  |  | Cropping sys. | 0.34 | 1 | 5.57E-01 |  |
|  |  | Variety:Cropping sys. | 0.04 | 2 | 9.78E-01 |  |
|  | Field 2022-2023 | Variety | **90.11** | **2** | **2.00E-16** | ******* |
|  |  | Cropping sys. | **4.86** | **1** | **2.74E-02** | ***** |
|  |  | Variety:Cropping sys. | 3.68 | 2 | 1.59E-01 |  |
|  | Field 2023-2024 | Variety | **69.72** | **2** | **7.24E-16** | ******* |
|  |  | Cropping sys. | 1.39 | 1 | 2.39E-01 |  |
|  |  | Variety:Cropping sys. | **19.44** | **2** | **6.01E-05** | ******* |
| Stem height | Glasshouse | **Variety** | **5.47** | **2** | **6.48E-02** | **.** |
|  |  | **Cropping sys.** | **5.18** | **1** | **2.28E-02** | ***** |
|  |  | Variety:Cropping sys. | 0.11 | 2 | 9.48E-01 |  |
|  | Field 2022-2023 | **Variety** | **30.88** | **2** | **1.97E-07** | *** |
|  |  | **Cropping sys.** | **20.18** | **1** | **7.06E-06** | *** |
|  |  | **Variety:Cropping sys.** | **6.64** | **2** | **3.61E-02** | * |
|  | Field 2023-2024 | **Variety** | **8.83** | **2** | **1.21E-02** | * |
|  |  | Cropping sys. | 0.46 | 1 | 4.99E-01 |  |
|  |  | **Variety:Cropping sys.** | **13.53** | **2** | **1.16E-03** | ****** |
| Branching | Glasshouse | Variety | 1.34 | 2 | 5.13E-01 |  |
|  |  | **Cropping sys.** | **4.96** | **1** | **2.59E-02** | ***** |
|  |  | Variety:Cropping sys. | 0.67 | 2 | 7.14E-01 |  |
|  | Field 2022-2023 Zero-inflated model | Variety | 14.44 | 2 | 7.31E-04 | *** |
|  |  | **Cropping sys.** | **17.20** | **1** | **3.36E-05** | *** |
|  |  | Variety:Cropping sys. | 0.61 | 2 | 7.37E-01 |  |
|  | Field 2022-2023 count model | **Variety** | **11.99** | **2** | **2.49E-03** | ** |
|  |  | Cropping sys. | 1.88 | 1 | 1.70E-01 |  |
|  |  | Variety:Cropping sys. | 3.16 | 2 | 2.06E-01 |  |
|  | Field 2023-2024 Zero-inflated model | Variety | 0.50 | 2 | 7.79E-01 |  |
|  |  | Cropping sys. | 0.00 | 1 | 9.96E-01 |  |
|  |  | Variety:Cropping sys. | 0.64 | 2 | 7.25E-01 |  |
|  | Field 2023-2024 count model | Variety | 2.34 | 2 | 3.11E-01 |  |
|  |  | Cropping sys. | 0.00 | 1 | 9.57E-01 |  |
|  |  | Variety:Cropping sys. | 0.31 | 2 | 8.55E-01 |  |
| Thousand kernel weight | Field 2022-2023 | Variety | 2.00 | 2 | 3.69E-01 |  |
|  |  | Cropping sys. | 0.03 | 1 | 8.60E-01 |  |
|  |  | Variety:Cropping sys. | 0.32 | 2 | 8.54E-01 |  |
|  | Field 2023-2024 | Variety | 3.14 | 2 | 2.08E-01 |  |
|  |  | Cropping sys. | 0.00 | 1 | 9.69E-01 |  |
|  |  | Variety:Cropping sys. | 2.51 | 2 | 2.86E-01 |  |
| Seeds per square meter | Field 2022-2023 | Variety | 3.53 | 2 | 1.71E-01 |  |
|  |  | **Cropping sys.** | **3.12** | **1** | **7.73E-02** | **.** |
|  |  | Variety:Cropping sys. | 0.01 | 2 | 9.94E-01 |  |
|  | Field 2023-2024 | Variety | 0.44 | 2 | 8.04E-01 |  |
|  |  | Cropping sys. | 0.09 | 1 | 7.59E-01 |  |
|  |  | Variety:Cropping sys. | 4.36 | 2 | 1.13E-01 |  |
| Yield | Field 2022-2023 | Variety | 1.48 | 2 | 4.78E-01 |  |
|  |  | **Cropping sys.** | **4.76** | **1** | **2.92E-02** | * |
|  |  | Variety:Cropping sys. | 0.06 | 2 | 9.71E-01 |  |
|  | Field 2023-2024 | Variety | 0.86 | 2 | 6.51E-01 |  |
|  |  | Cropping sys. | 0.12 | 1 | 7.31E-01 |  |
|  |  | **Variety:Cropping sys.** | **5.08** | **2** | **7.88E-02** | **.** |

**Table S4.** Summary table showing the output of statistical models applied to oilseed rape insect pest incidence and damage, analyzing the effects of two factors (variety and cropping system) and their interaction across the two field experiment campaigns.

| **Response** | **Experiment** | **Factor** | **Chisq** | **Df** | **P-value** |  |
| --- | --- | --- | --- | --- | --- | --- |
| Shotholes on cotyledons | Field 2022-2023 | Variety | 0.94 | 2 | 6.24E-01 |  |
|  |  | Cropping sys. | 0.64 | 1 | 4.23E-01 |  |
|  |  | Variety:Cropping sys. | 0.07 | 2 | 9.68E-01 |  |
|  | Field 2023-2024 | Variety | 1.11 | 2 | 5.74E-01 |  |
|  |  | Cropping sys. | 1.87 | 1 | 1.71E-01 |  |
|  |  | Variety:Cropping sys. | 1.66 | 2 | 4.36E-01 |  |
| *Psylliodes chrysocephala* larval infestation | Field 2022-2023 | Variety | 3.32 | 2 | 1.90E-01 |  |
|  |  | Cropping sys. | 0.19 | 1 | 6.65E-01 |  |
|  |  | Variety:Cropping sys. | 1.86 | 2 | 3.95E-01 |  |
|  | Field 2023-2024 | **Variety** | **23.69** | **2** | **7.18E-06** | ******* |
|  |  | Cropping sys. | 1.74 | 1 | 1.87E-01 |  |
|  |  | **Variety:Cropping sys.** | **6.09** | **2** | **4.77E-02** | ***** |
| *Ceutorhynchus napi* oviposition on stems | Field 2022-2023 zero-inflated model | Variety | 0.59 | 2 | 7.44E-01 |  |
|  |  | Cropping sys. | 0.00 | 1 | 9.54E-01 |  |
|  |  | Variety:Cropping sys. | 0.00 | 2 | 1.00E+00 |  |
|  | Field 2022-2023 count model | Variety | 1.35 | 2 | 5.08E-01 |  |
|  |  | Cropping sys. | 2.21 | 1 | 1.37E-01 |  |
|  |  | Variety:Cropping sys. | 3.80 | 2 | 1.50E-01 |  |
|  | Field 2023-2024 zero-inflated model | Variety | 0.12 | 2 | 9.40E-01 |  |
|  |  | Cropping sys. | 0.14 | 1 | 7.04E-01 |  |
|  |  | Variety:Cropping sys. | 0.22 | 2 | 8.97E-01 |  |
|  | Field 2023-2024 count model | Variety | 2.29 | 2 | 3.18E-01 |  |
|  |  | **Cropping sys.** | **3.09** | **1** | **7.89E-02** | **.** |
|  |  | Variety:Cropping sys. | 2.16 | 2 | 3.40E-01 |  |
| *Brassicogethes aeneus* on inflorescence | Field 2022-2023 | **Variety** | **8.16** | **2** | **1.69E-02** | ***** |
|  |  | **Cropping sys.** | **8.16** | **1** | **4.29E-03** | ****** |
|  |  | Variety:Cropping sys. | 3.55 | 2 | 1.69E-01 |  |
|  | Field 2023-2024 zero-inflated model | Variety | 4.25 | 2 | 1.20E-01 |  |
|  |  | Cropping sys. | 0.08 | 1 | 7.74E-01 |  |
|  |  | Variety:Cropping sys. | 0.74 | 2 | 6.92E-01 |  |
|  | Field 2023-2024 count model | Variety | 2.94 | 2 | 2.30E-01 |  |
|  |  | Cropping sys. | 0.45 | 1 | 5.03E-01 |  |
|  |  | Variety:Cropping sys. | 0.28 | 2 | 8.71E-01 |  |

**Table S5.** Summary table showing the output of statistical models applied to microclimatic response, analyzing the effects of cropping system across the glasshouse and field experiment.

| **Response** | **Experiment** | **Factor** | **F/Khi2** | **Df** | **P-value** |  |
| --- | --- | --- | --- | --- | --- | --- |
| Temperature under canopy | Glasshouse | Cropping sys. | 15.05 | 1 | 2.12E-04 | *** |
|  | Field 2022-2023 | Cropping sys. | 14.76 | 1 | 2.68E-04 | *** |
|  | Field 2023-2024 | Cropping sys. | 8.06 | 1 | 7.00E-03 | ** |
| PAR under canopy | Glasshouse | Cropping sys. | 4.85 | 1 | 2.76E-02 | * |
|  | Field | Cropping sys. | 9.06 | 1 | 8.00E-03 | ** |
| PAR above OSR canopy | Glasshouse | Cropping sys. | 16.60 | 1 | 4.62E-05 | *** |
|  | Field | Cropping sys. | 10.97 | 1 | 9.29E-04 | *** |
| R:FR under canopy | Glasshouse | Cropping sys. | 6.43 | 1 | 1.12E-02 | * |
|  | Field | Cropping sys. | 5.39 | 1 | 3.40E-02 | * |
| R:FR above OSR canopy | Glasshouse | Cropping sys. | 14.00 | 1 | 1.83E-04 | *** |
|  | Field | Cropping sys. | 12.79 | 1 | 3.49E-04 | *** |

**Figure S1.** Morphological traits and oilseed rape crop characteristics of the variety Feliciano when intercropped with faba beans or in monocropping. The blue background in the graphs indicate the wintering or winter period in the experiments. A- Cumulated number of leaves during the growing period -Expressed in cumulated degree days- in the glasshouse (GH) experiment. B- Estimated Marginal Means of the number of leaves at BBCH 17 for the GH and 2022-2023 field experiment and later in the season for the 2023-2024 field experiment. C- Estimated Marginal Means of single plant dry biomass after winter for the field experiments. D- Elongation of the stem in cm after the wintering period to the blooming period in the GH experiment. E- Same measurement for the field experiment in the cropping season 2022-2023. F- Same measurement for the field experiment in the cropping season 2023-2024. G- Predicted counts and zero-inflation probabilities for branching of intercropped and monocropped OSR under the three experimental conditions. The bar plot with the left y-axis represents the predicted count of branching. The zero-inflation probability is shown as points, with the right y-axis. H- Estimated Marginal Means of OSR yield in kg/acre for the two field experiments. Statistical significance: “.” for p ≤ 0.1, “*” for p ≤ 0.05, “**” for p ≤ 0.01, “***” for p ≤ 0.001, and “NS” for not significant (p > 0.05), in case of no indication no statistical analysis was applied.

Branching_NB

**Figure S2.** Pearson correlation matrix of oilseed rape (OSR) morphological traits, insect pest incidence and damage, crop characteristics, yield components, and final yield. The matrix is based on per-plot data from the two field experiment campaigns described in Chapter IV. Dots indicate statistically significant correlations (P < 0.05); blue shades represent positive correlations; orange shades represent negative correlations. Pearson correlation coefficients (R) are displayed within each significant dot. Morphological traits: Leaves_NB (number of leaves at BBCH 17), Collar_D (collar diameter before winter), Biomass (dry OSR biomass at BBCH 30), Stem_elongation (stem elongation at BBCH 32–34), Stem_height (main stem height at BBCH 80 in 2023, BBCH 61 in 2024), Branching_NB (number of branches at BBCH 80 in 2023, BBCH 61 in 2024). Insect pest incidence and damage: Shotholes_cotyledons_NB (feeding holes by Phyllotreta spp. on cotyledons, BBCH 12), Larval_infestation (Psylliodes chrysocephala larvae per plant, BBCH 30), Oviposition_NB (Ceutorhynchus napi oviposition punctures per stem, BBCH 32–34), Pollenbeetle_NB (Brassicogethes aeneus adults per inflorescence, BBCH 57). Crop characteristic: Density_OSR_feb (plant density per m² in February). Yield components: Thousand_kernel_weight (weight of 1,000 seeds), Seeds_per_smeter (calculated number of seeds per square meter). Yield: Yield (final OSR seed yield per plot).
